# POMs‐GibbsNet: A Deep Neural Network for the Prediction of Thermodynamic Parameters of Polyoxometalates

**DOI:** 10.1002/jcc.70510

**Published:** 2026-10-21

**Authors:** Jordi Buils, Carles Bo, David Balcells

**Affiliations:** ^1^ Institute of Chemical Research of Catalonia (ICIQ) Tarragona Spain; ^2^ Departament de Química Física I Inorgànica Universitat Rovira i Virgili Tarragona Spain; ^3^ Hylleraas Centre for Quantum Molecular Sciences, Department of Chemistry University of Oslo Oslo Norway

**Keywords:** AABBA, neural network, POMSimulator, POMs‐GibbsNet

## Abstract

Polyoxometalates (POMs) are versatile metal–oxo clusters with remarkable structural diversity and applications spanning catalysis, energy storage, and materials science. However, accurately predicting their thermodynamic properties remains computationally demanding due to the large system sizes and the cost of density functional theory (DFT) frequency calculations required to obtain Gibbs free energies. In this work, we present POMs‐GibbsNet, a deep neural network designed to predict the thermal free energy correction (G−E) of polyoxometalates by combining graph‐based molecular representations with DFT‐derived descriptors. Molecular structures were encoded using variants of the AABBA autocorrelation graph kernel, which captures atom–atom, bond–bond, and atom–bond correlations together with quantum‐derived features including QTAIM descriptors. POMs‐GibbsNet was trained on a dataset of 1419 POM structures spanning 19 chemical systems to obtain good results ($R^{2}=0.993$ and RMSE = 1.9 kcal · mol^−1^. To evaluate the performance, a benchmark against 10 classical regressors was performed. Additionally, an uncertainty study was added to the methodology. External validation using POMSimulator reaction networks comprising over 10,819 reactions shows excellent agreement between predicted and DFT reaction energies ($R^{2}>0.99$), with errors typically within a few kcal mol^−1^. Error cancellation was also studied during this step. Notably, simplified atom–atom autocorrelation descriptors already provide robust performance while reducing model complexity. These results demonstrate that POMs‐GibbsNet enables efficient estimation of thermodynamic corrections in POM systems, providing a powerful framework for accelerating the computational exploration of polyoxometalate speciation and self‐assembly across large chemical spaces.

## Graphical abstract

Polyoxometalate thermochemistry is often limited by the high cost of DFT vibrational frequency calculations. POMs‐GibbsNet uses molecular structure, electronic, and QTAIM descriptors to predict thermal Gibbs free‐energy corrections without calculating the Hessian. The model preserves reaction thermodynamics and enables substantially faster exploration of POM reaction networks, with the computational advantage increasing for larger clusters.

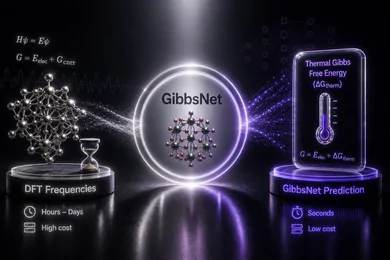

## 1 Introduction

Polyoxometalates represent a remarkably versatile class of molecular metal–oxo clusters, comprising early transition metals in high oxidation states (typically $Mo^{6+}$, $W^{6+}$, $V^{5+}$, $Nb^{5+}$, $Ta^{5+}$) [1, 2] interconnected by oxo ligands into discrete nanoscale architectures spanning 1 to 6 nm. First systematically explored in the late 19th century by Berzelius and elaborated through pioneering works in the 20th century—such as Keggin's structural elucidation in 1933—these polynuclear anions have surged in relevance over the past decades due to their unprecedented structural diversity [2, 3, 4, 5]. This encompasses iconic archetypes like Keggin ${\left[XM_{12}O_{40}\right]}^{n-}$, Dawson ${\left[X_{2}M_{18}O_{62}\right]}^{m-}$, Anderson–Evans ${\left[XM_{6}O_{24}\right]}^{p-}$, and Lindqvist ${\left[M_{6}O_{19}\right]}^{q-}$, alongside lacunary derivatives (with metal vacancies for functionalization), [4, 6, 7, 8] transition‐metal‐substituted variants (e.g., lacunary Keggins with Co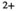, Cu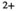, Fe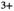), organo‐functionalized hybrids, and even giant supramolecular assemblies exceeding 100 metal centers [6, 9, 10].

Their pH‐responsive speciation, dynamic self‐assembly pathways, and rich multi‐electron redox chemistry in aqueous media—spanning multiple accessible potentials—enable transformative applications across homogeneous and heterogeneous catalysis [11, 12, 13] (e.g., selective oxidations of alkanes, alkenes, and sulfides using $PW_{12}O_{40}^{3-}$), energy storage [12, 14] (pseudocapacitors with capacitance >500 F g^−1^, lithium‐ion battery cathodes), medicine [15, 16, 17, 18] (antiviral agents against HIV, influenza, and COVID‐19; anticancer via ROS (reactive oxygen species) generation and DNA binding), photovoltaics, and advanced functional materials [4, 7, 19] such as luminescent probes and sensors.

Yet, the intricate mechanisms governing their assembly—from mononuclear precursors via condensation to thermodynamically favored clusters—their inherent conformational flexibility (e.g., Jahn–Teller distortions in vanadium POMs), and pronounced sensitivity to counterions (e.g., alkali cations templating speciation), pH, and solvent effects pose formidable barriers to both experimental synthesis (low yields, purification challenges) [9, 20, 21] and traditional computational modeling [1, 17, 22] (e.g., DFT's prohibitive scaling for >100‐atom systems, inadequate solvation models, and sampling of protonation or tautomer states) [23]. Accurately predicting key equilibrium constants—such as formation constants $K_{f}$, acid dissociation constants $pK_{a}$, and protonation sequences—is essential for constructing speciation diagrams, optimizing synthetic conditions, and elucidating reactivity [21, 24, 25]. However, this remains notoriously challenging due to sparse experimental data and modeling inaccuracies, underscoring an urgent need for innovative predictive tools such as machine learning [26].

POMSimulator [25] represents a pioneering computational platform tailored for modeling the speciation, self‐assembly pathways, and thermodynamic stability of polyoxometalates under varying pH, counterion, and solvent conditions. By integrating density functional theory(DFT) calculations with statistical post‐processing [21, 24], it circumvents the prohibitive computational costs of explicit high‐level quantum modeling for large clusters, enabling the construction of comprehensive Pourbaix diagrams and equilibrium speciation maps essential for synthetic design and reactivity prediction.

Recent advancements in POMSimulator feature a universal scaling [27] protocol that applies a linear correction to refine DFT‐derived formation constants ($K_{f}$).

$$\log K_{f}^{\text{scaled}}=m\cdot \log K_{f}^{\text{DFT}}+b$$

Here, the slope m exhibits remarkable system‐independence across diverse POM families, reflecting consistent systematic DFT biases in metal–oxo condensation energetics. The intercept b, however, is dynamically predicted through a multilinear regression (MLR) model trained on an extensive array of statistical descriptors—such as mean absolute deviations, variance in bond orders, and cluster size metrics—extracted from unscaled DFT outputs. This hybrid approach mitigates errors from functional choice and solvation approximations while enabling reliable extrapolation to unexplored chemical space.

Building on these hybrid DFT–statistical methods exemplified by POMSimulator, machine learning emerges as a powerful paradigm to tackle longstanding challenges in polyoxometalate thermodynamics [26]. While DFT excels at providing mechanistic insights into metal–oxo bonding and condensation energetics, it struggles with scalability for clusters exceeding dozens of metal centers, inaccuracies in explicit solvation models beyond continuum approximations like COSMO, and the combinatorial explosion of configurational sampling required to account for protonation isomers, counterion pairings, and dynamic assembly pathways [28]. Machine learning circumvents these hurdles by constructing data‐driven surrogate models that learn complex structure–property relationships [29, 30] from high‐fidelity quantum datasets, delivering predictions orders of magnitude faster while enabling uncertainty quantification and active learning strategies [31].

Key to this advancement are molecular representations and architectures tailored for inorganic systems [32]. Graph‐based methods, [33] such as message‐passing neural networks or kernel encodings, effectively capture the topological intricacies of POMs, including the symmetries of the addenda metal‐oxo framework (e.g., Keggin α/β isomers), lacunary defects, and polyhedral connectivity. Models trained on specialized datasets such as tmQM [34] or Hessian QM9 [35] facilitate accurate inference of vibrational free energies, protonation constants ($pK_{a}$), formation equilibria ($K_{f}$), and redox potentials.

For POMs specifically, machine learning bridges the gap between computationally demanding DFT pipelines and experimental imperatives, as demonstrated by POMSimulator's statistical post‐processing for scaling formation constants. These approaches integrate seamlessly with global optimization techniques, such as genetic algorithms or Bayesian optimization, to explore vast chemical spaces for stable POM variants.

A critical aspect of applying machine learning to POMs is generating effective molecular fingerprints that capture their topological complexity, metal–oxo connectivity, and conformational flexibility. Common approaches include:

- *Traditional fingerprints*: Bit‐vector methods such as Extended Connectivity Fingerprints (ECFP/Morgan) [36] or Daylight fingerprints, which encode substructures but struggle with inorganic symmetries and multicenter bonds.
- *Descriptor‐based methods*: Physicochemical descriptors from tools like RDKit or Dragon, aggregating atomic properties but often lacking graph topology.
- *Local environment descriptors*: Smooth Overlap of Atomic Positions or Atom‐Centered Symmetry Functions, ideal for periodic solids but less suited for discrete clusters.
- *Graph neural networks*: Message‐passing schemes (e.g., SchNet, [37] DimeNet [38, 39]) that learn iterative embeddings from node and edge features.

Among these approaches, graph‐based kernels such as AABBA [40] excel for transition metal complexes, as well as for other clusters such as MOFs [41], hinting at promising applications for POMs.

The AABBA graph kernel encodes molecular structures as fixed‐length vectors through atom–atom, bond–bond, and atom–bond autocorrelations, systematically capturing both local chemical environments and global topological features essential for POMs. Unlike atom‐only representations, AABBA incorporates bond order and path information, enabling superior performance in predicting energy barriers, bond distances, and thermodynamic properties. Its modular design also allows dimensionality reduction for interpretability, making it particularly suitable for integration with DFT features in models such as POMs‐GibbsNet.

POMs‐GibbsNet is a deep neural network designed to predict POM thermodynamic parameters by fusing DFT‐derived features with advanced molecular representations. Trained on a curated dataset of 19 diverse POM systems, represented in Figure 1. From simple IPA [21, 42, 43] systems: Mo, W, V, Nb and Ta; typical HPAs:SiW, PW, PMo [24], or AsMo; other kind of mixed poms: AsMoW, AsTaW, AsW, PMoW, PTaW, SiMo, SiMoW, SiTaW, VMo, VW. Machine learning of thermodynamic quantities has a well‐established precedent for inorganic solids and molecular systems, from formation‐energy surrogates for crystalline materials [29] to message‐passing models that reach chemical accuracy on molecular energetics [32] and, more recently, to dedicated free‐energy and thermochemical property predictors [30]. Parallel progress in descriptor‐based screening of inorganic and coordination materials [44, 45], in machine‐learning‐accelerated property prediction [46] and in anomaly detection within chemical datasets [47] shows that carefully engineered descriptors remain competitive with learned representations in the low‐data regime that POM chemistry inevitably occupies The contribution of this work is therefore not a new architecture but a validated, uncertainty‐aware, provenance‐audited descriptor‐plus‐surrogate workflow that removes the frequency calculation from an automated reaction‐network generator, together with an explicit characterization of where it fails.

**FIGURE 1:**
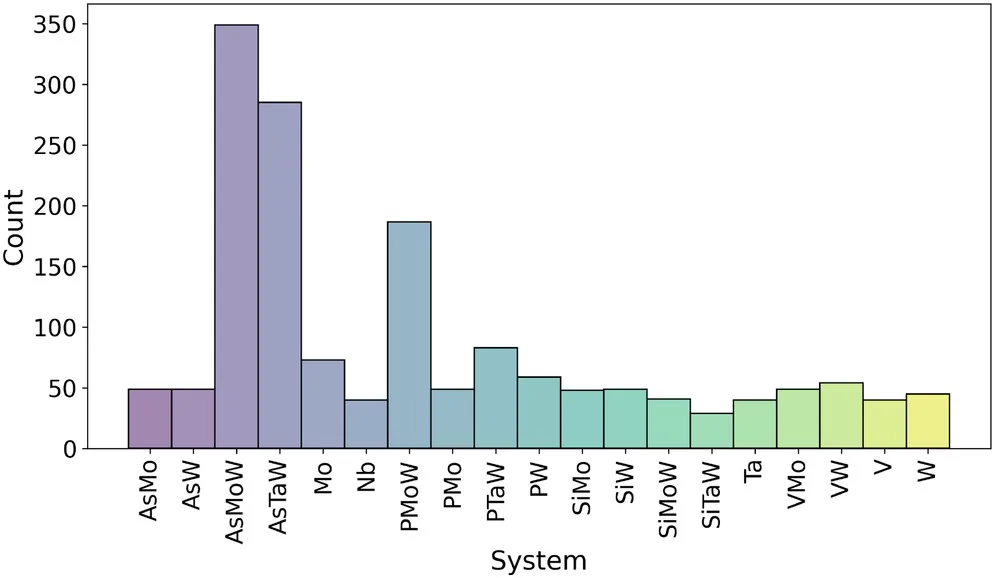
Bar plot for the number of polyoxometalate species in the dataset, grouped by system, with a global count of 1419 molecular graphs.

## 2 Methodology

### 2.1 DFT

All DFT calculations were performed using the SCM ADF 2019.14 [48] package with the PBE [49, 50] functional and TZP basis set for all elements. Geometry optimizations and frequency calculations incorporated the Zero‐Order Regular Approximation (ZORA) [51, 52] to account for relativistic effects. Water solvent effects were modeled using the COSMO solvation model implemented in ADF with Klamt's default radii [53]. Stationary points were characterized through analytical frequency analysis, yielding ground‐state Gibbs free energies at 1 atm and 298.15 K.

Quantum Theory of Atoms in Molecules (QTAIM) [54, 55, 56] analysis was performed to rigorously define molecular connectivity and bonding. QTAIM, developed by Richard Bader, employs the topology of the electron density distribution to partition molecules into atomic basins separated by zero‐flux surfaces and to identify bond critical points (BCPs) along bond paths.

At these BCPs, key topological parameters were extracted to characterize bond nature and strength in polyoxometalates. The electron density [57, 58] ρ(r) quantifies electron accumulation at the BCP, with higher values indicating stronger bonding interactions. The Laplacian [58, 59, 60] $\nabla^{2}\rho (r)$ distinguishes interaction types: negative values signify covalent shared interactions, while positive values denote closed‐shell ionic or hydrogen bonding.

Bond ellipticity [61, 62] measures deviations from cylindrical symmetry and reveals π‐character in bonds.

Abramov's [63] energy densities at BCPs include the local kinetic energy density $G_{b}>0$, potential energy density $V_{b}<0$, and total energy density

$$H_{b}=G_{b}+V_{b}$$

where $H_{b}<0$ indicates covalent character and correlates with bond dissociation energy.

Recent work has highlighted the growing relevance of QTAIM‐derived descriptors in machine learning frameworks for complex chemical systems. In particular, high‐throughput implementations of QTAIM enable the systematic generation of electron density‐based features that can be directly integrated into graph neural network architectures for molecular and reaction property prediction [64]. These studies demonstrate that QTAIM features consistently improve predictive performance across diverse datasets, while also enhancing generalization to out‐of‐domain chemical spaces and low‐data regimes. In parallel, multilevel QTAIM‐enriched graph neural networks have shown that incorporating ab initio topological descriptors across different levels of theory leads to improved robustness and accuracy in predicting properties of transition metal complexes, particularly in extrapolative scenarios involving unseen compositions or limited training data [65]. Together, these advances reinforce the value of QTAIM as a physically grounded and transferable descriptor set for data‐driven modeling, supporting its incorporation into the present framework.

The computational advantage of POMs‐GibbsNet arises from replacing the expensive DFT frequency calculation with a workflow based on geometry optimization, single‐point electronic structure calculations, QTAIM analysis, descriptor construction, and neural‐network inference. A stage‐resolved computational cost analysis (Supporting Information) shows that, once an optimized geometry is available, the descriptor‐generation and prediction workflow requires less than 0.1% of the computational cost of a Hessian calculation. Even when the geometry optimization is included, the complete workflow remains approximately one order of magnitude faster than performing a full vibrational analysis. Owing to the more favorable scaling of the retained computational steps, the relative speed‐up increases with system size, making the proposed approach particularly attractive for the large polyoxometalate clusters targeted by automated reaction‐network generation. A detailed description of the cost model, scaling analysis, and timing estimates is provided in the Supporting Information.

### 2.2 AABBA

The AABBA graph kernel, as described by Morán et al. [40] was employed to generate fixed‐length vector representations of polyoxometalate molecular graphs. This kernel computes autocorrelation functions over atom–atom, bond–bond, and atom–bond pairs up to a defined path depth, encoding both local chemical environments and global structural motifs.

Polyoxometalates feature multiple metal centers and therefore require the “full” approximation. The maximum autocorrelation depth was set to half of the median maximum depth across the dataset. Three arithmetic operations (multiplication, sum, and division) were tested independently to determine the highest‐performing configuration.

These autocorrelation descriptors were augmented with node, edge, and whole‐graph properties. Node features included atomic number, node degree, covalent radius, electronegativity, and DFT‐derived multipole charges, $Q_{m}$, $Q_{d}$, $Q_{q}$. Edge features incorporated QTAIM properties [61, 63, 66] 0 (ρ, $\nabla^{2}\rho$, the ellipticity ϵ, the diamond ◇, metallicity indices, and Abramov energy densities $G_{b}$, $V_{b}$, $H_{b}$), together with bond distance and estimated bond order.

The whole‐graph features included the total number of atoms and electrons, molecular charge, molecular weight, HOMO and LUMO energies, HOMO–LUMO gap. All seven features are available from the geometry optimization and the subsequent single‐point calculation. Neither the Electronic nor the Gibbs free energies are used as input features.

The graphs were labeled using the difference between Gibbs free energy G and electronic energy E:

$$\Delta G_{\text{thermal}}=G-E$$

Nine feature vectors are used in this work, and their dimensions follow directly from the above: the atom–atom‐only representation (AA, 20), the full autocorrelation kernel (AABBA, 108 features), and the extended kernel including the whole‐graph block (extended AABBA, 115 features).

### 2.3 POMs‐GibbsNet Architecture and Training Hyperparameters

Hyperparameter optimization was performed with the Optuna [67] library using the default TPE sampler, in a persisted study of 100 completed trials. The objective was the coefficient of determination on the validation split, maximized; a median pruner (one start‐up trial, five warm‐up epochs, five‐epoch evaluation interval) terminated unpromising trials. The search space comprised the hidden width (32–128 neurons, Step 1), the number of hidden layers (2–12), the activation function (ELU, ReLU or LeakyReLU), the use of batch normalization, the learning rate (log‐uniform, 10^−4^–10^−2^) and the batch size (16–128, Step 2). Optimization used the extended AABBA representation with the multiplication operator, resampled by label‐grouped five‐fold cross‐validation with 15% of each training partition reserved as the validation set. The test partition was never used at any point in model selection, either for the network or for the classical baselines, each of which received an independent 40‐trial optimization under the identical protocol.

The selected configuration (best validation $R^{2}$ = 0.9982) is an input layer of 108 neurons (AABBA) or 115 neurons (extended AABBA), The final architecture consisted of five hidden layers. The first hidden layer contained 105 neurons, and the width of each subsequent hidden layer was reduced linearly, culminating in a single‐neuron output layer. Each layer was activated using ELU and no batch normalization was performed. Training minimized the mean squared error with the Adam optimizer for 100 epochs at a learning rate of 1.4214 × 10^−3^ and a batch size of 18, with the learning rate reduced on plateau (patience five epochs). No weight decay and no early stopping were applied.

### 2.4 POMSimulator

POMSimulator is a computational framework specifically designed to address the challenging problem of polyoxometalate (POM) speciation and self‐assembly in aqueous solution. Unlike conventional approaches that rely on manual construction of reaction schemes, POMSimulator automates the generation of extensive chemical reaction networks directly from DFT data. By systematically identifying possible transformations between molecular species—including condensation, protonation, and addition reactions—the method constructs large‐scale reaction networks that capture the complex multi‐equilibria processes governing POM formation. This automated strategy enables the exploration of thousands of chemically plausible reactions, providing a comprehensive description of the underlying thermodynamic landscape.

A central feature of POMSimulator is its ability to translate these reaction networks into speciation models, which are subsequently solved to obtain concentration–pH diagrams and phase diagrams. The methodology relies on statistical sampling of a large number of speciation models, allowing the extraction of averaged predictions together with associated uncertainties. This approach has proven particularly powerful for systems such as phosphomolybdate clusters, where it successfully reproduces experimentally observed speciation behavior and reveals key intermediates involved in self‐assembly pathways. In addition, the large‐scale nature of the generated datasets enables the identification of dominant reaction pathways and building blocks, offering mechanistic insights that are difficult to access experimentally.

Despite its predictive capabilities, the accuracy of POMSimulator is inherently linked to the quality of the underlying DFT‐derived thermodynamic data. Systematic errors in calculated formation constants, particularly those associated with solvation effects and entropy contributions, can propagate through the reaction network and affect speciation predictions. To address this limitation, recent developments have introduced a universal scaling approach that corrects DFT‐derived formation constants through a linear transformation. In this framework, the slope of the scaling relationship is found to be largely system‐independent, while the intercept can be predicted using a MLR model trained on statistical descriptors of the dataset. This hybrid DFT–statistical methodology significantly improves the agreement between predicted and experimental equilibrium constants, enabling reliable predictions even for systems with limited experimental data.

Within this context, POMSimulator provides an ideal platform for validating machine learning models such as POMs‐GibbsNet. By generating large and diverse reaction networks, it allows the assessment of predicted thermodynamic quantities across a wide range of chemical transformations and molecular systems. The integration of machine learning corrections into the POMSimulator workflow further enhances its efficiency by replacing computationally expensive DFT‐derived thermal contributions with rapid surrogate predictions. As a result, the combined POMSimulator–POMs‐GibbsNet framework enables high‐throughput exploration of polyoxometalate speciation and self‐assembly processes, opening new avenues for the rational design and discovery of complex metal–oxo clusters.

## 3 Results and Discussion

### 3.1 POMs‐GibbsNet Performance

To evaluate the effectiveness of the POMs‐GibbsNet architecture and determine the most suitable molecular representation for the prediction task, several variants of the AABBA autocorrelation vector were systematically tested. These representations differ in their level of structural detail and dimensionality, allowing an assessment of how different types of chemical information contribute to predictive accuracy.

The most comprehensive representation consisted of the full AABBA graph kernel augmented with whole‐graph descriptors, resulting in a feature vector containing 126 attributes. The use of the complete AABBA kernel was motivated by previous work by Morán et al., which demonstrated that this representation performs particularly well for transition metal complexes by capturing both local chemical environments and long‐range structural correlations within the molecular graph. By combining atom–atom, bond–bond, and atom–bond autocorrelations, the AABBA representation provides a rich description of the connectivity patterns and bonding environments characteristic of polyoxometalate clusters.

Within this feature space, three arithmetic operations were explored for constructing the autocorrelation descriptors: multiplication, division, and summation. These operations control how the atomic and bond properties propagate along paths in the molecular graph, thereby affecting the sensitivity of the representation to different structural motifs. Although the performance according to statistical metrics ($R^{2}$, RMSE and MAE) was quite similar for the three configurations, multiplication was slightly better and therefore selected for subsequent hyperparameter optimization and final model training (Figure 2).

**FIGURE 2:**
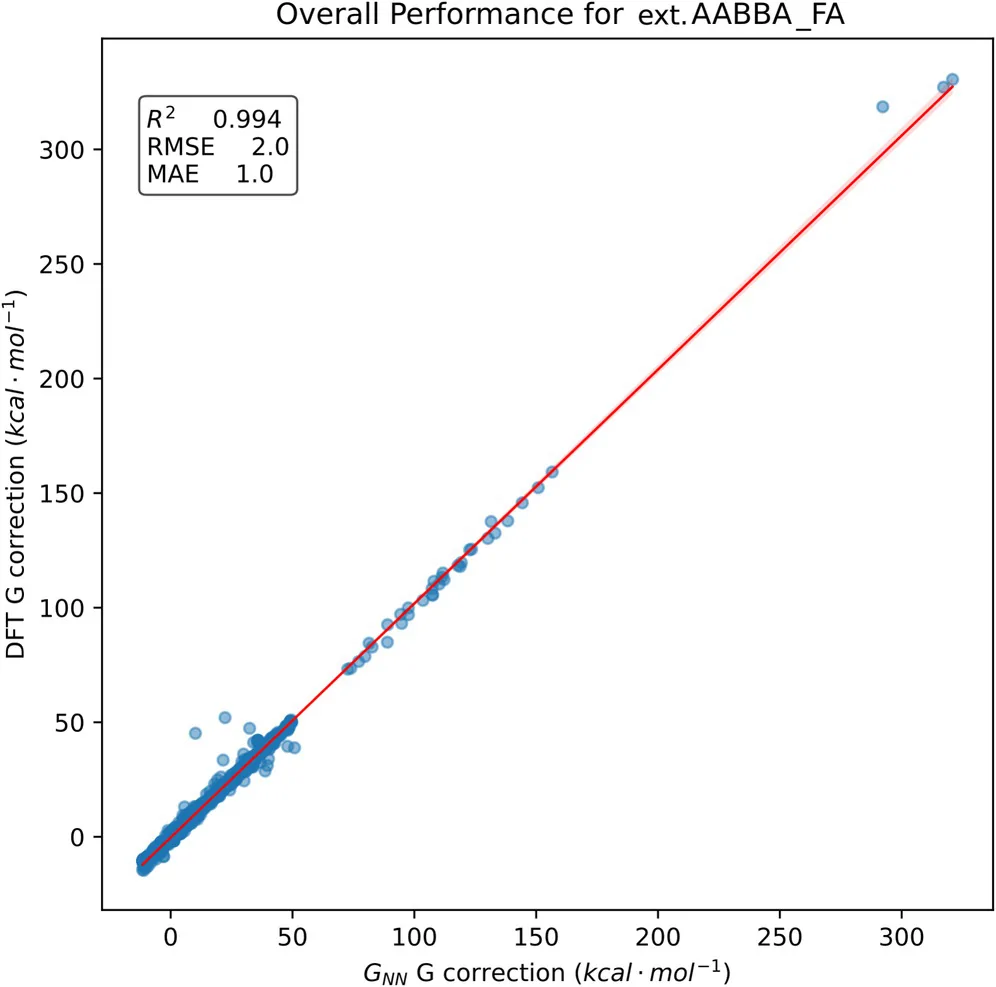
POMs‐GibbsNet predictions vs DFT pair plots using AABBA autocorrelation vector plus whole graph features using the product operation.

In order to investigate whether a reduced feature space could maintain predictive accuracy while improving computational efficiency and interpretability, the representation was subsequently simplified by removing the additional whole‐graph descriptors. This reduced configuration consisted solely of the extended AABBA kernel (112 features), incorporating Atom–Atom, Bond–Bond, and Atom–Bond autocorrelations. As in the previous case, the three mathematical operations (multiplication, division, and sum) were evaluated independently.

The resulting models displayed performance trends similar to those observed for the larger representation. Like before, sum and multiplication operations perform similarly and clearly better than division.

Finally, a minimal baseline representation was evaluated in order to determine the extent to which predictive performance depends on the inclusion of bond‐level information. In this simplified scenario, only Atom–Atom autocorrelations were used, producing a 20‐feature vector that was combined with the three mathematical operations. Despite the drastic reduction in feature dimensionality, the resulting models still achieved comparable performance levels, indicating that even relatively simple graph‐based descriptors can capture important aspects of the structural variability within the dataset. A quantitative comparison of the different representations is provided in Table 1.

**TABLE 1:** Performance of different autocorrelation (AC) representations and operations after model optimization.

Descriptor	Operation	$R^{2}$	RMSE	MAE
AA	FA	0.994	2.0	1.2
AA	FR	0.983	3.2	2.2
AA	FS	0.996	1.7	1.1
AABBA	FA	0.996	1.5	1.0
AABBA	FR	0.989	2.6	1.8
AABBA	FS	0.995	1.7	1.1
ext. AABBA	FA	0.994	2.0	1.0
ext. AABBA	FR	0.994	2.0	1.3
ext. AABBA	FS	0.996	1.6	1.0

Taken together, these experiments show that the predictive accuracy obtained under a random train–test split is remarkably insensitive to the descriptor representation. Every combination of autocorrelation type and mathematical operator evaluated in Table 1 achieves $R^{2}\geq 0.983$ and MAE ≤2.2 kcal mol^−1^, while the difference between the best‐performing representation (extended AABBA with multiplication; $R^{2}=0.997$) and the simplest (AA with summation; $R^{2}=0.993$) corresponds to only 1.3 kcal mol^−1^ in RMSE.

The predictive performance reported above was obtained using a conventional random train–test split. Although this protocol provides a useful measure of interpolation accuracy, it can substantially overestimate predictive performance when chemically related structures appear in both the training and test sets. This is particularly relevant for polyoxometalates, where different protonation states, structural isomers, and metal‐substituted analogues often share highly similar structural and electronic characteristics. To assess the true generalization capability of POMs‐GibbsNet, two chemically informed validation protocols were additionally employed: leave‐one‐system‐out (LOSO) cross‐validation, in which all structures belonging to a given POM system were excluded from training, and leave‐one‐metal‐family‐out (LOMFO) cross‐validation, where entire metal families were withheld. The complete methodology and results are reported in the Supporting Information. Briefly, these analyses reveal that random train–test splits considerably overestimate transferability, with prediction accuracy deteriorating for previously unseen chemical systems, particularly small single‐metal isopolyanions.

In addition to evaluating predictive accuracy and transferability, we assessed the reliability of the model predictions through uncertainty quantification and applicability‐domain analyses. These results, including the calibration of Monte Carlo dropout uncertainties and the identification of out‐of‐domain systems, are presented in the Supporting Information.

The limited sensitivity to the descriptor representation does not imply that the neural‐network architecture is responsible for the observed predictive accuracy. A benchmark against ten classical machine‐learning regressors, using identical descriptors and train–test partitions.

At the present dataset size, the mapping between the descriptors and the thermal Gibbs free‐energy correction (G−E) appears to be close to linearly learnable, making linear and kernel‐based methods attractive choices for interpolation tasks. Nevertheless, POMs‐GibbsNet remains advantageous as a single differentiable model that naturally provides Monte Carlo dropout uncertainty estimates, scales without requiring kernel matrices, and is seamlessly integrated into the POMSimulator workflow.

### 3.2 MLR Baseline and Benchmark Against Classical Machine‐Learning Regressors

As an initial reference, a MLR model was developed using the same whole‐graph descriptors employed by POMs‐GibbsNet. These descriptors comprise global physicochemical properties obtained before the frequency calculation, including the number of atoms and electrons, molecular charge, molecular mass, solvent‐accessible surface area, molecular volume, frontier orbital energies (HOMO and LUMO), and the HOMO–LUMO gap. The target variable was the thermal Gibbs free‐energy correction (G−E).

The MLR model provides a simple and interpretable baseline for assessing the extent to which the relationship between these descriptors and the thermal correction can be described by a linear model. Although the linear model captures the overall trend, its predictive accuracy remains limited, yielding test‐set errors between approximately 6 and 10 kcal mol^−1^ depending on whether outliers are removed. These results indicate that linear combinations of global descriptors alone are insufficient to describe the complex thermodynamic behavior of polyoxometalates.

Because comparison against a single baseline provides only limited insight, we additionally benchmarked POMs‐GibbsNet against ten widely used regression algorithms using identical descriptors, train/test partitions, and evaluation metrics. The benchmark comprised ordinary least‐squares regression, Ridge, Lasso, Elastic Net, Support Vector Regression (SVR), Kernel Ridge Regression (KRR), Random Forest, Extra Trees, Gradient Boosting, and a multilayer perceptron (MLP). The resulting performance is summarized in Table 2 and illustrated in Figure 3.

**FIGURE 3:**
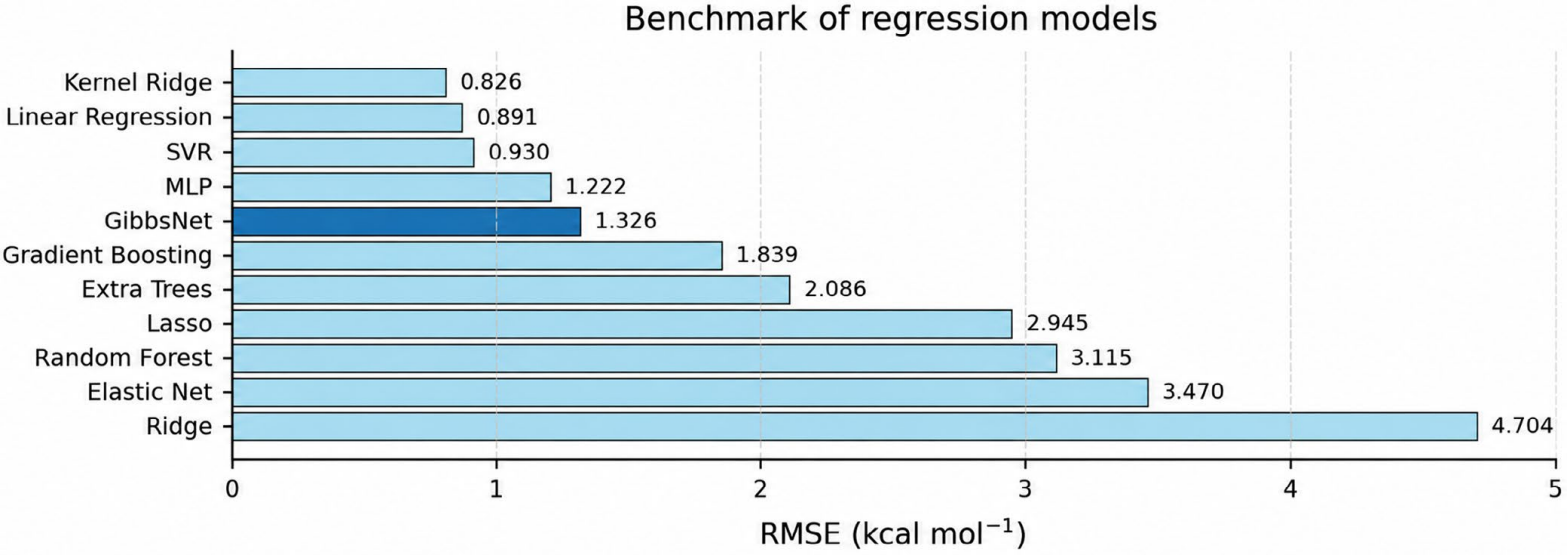
Benchmark of POMs‐GibbsNet and ten classical machine‐learning regression algorithms using identical descriptors and train/test partitions. Models are ranked according to the test‐set RMSE.

**TABLE 2:** Comparison between POMs‐GibbsNet and classical machine‐learning regressors using identical descriptors and train/test partitions.

Model	$R^{2}$	RMSE	MAE
Kernel Ridge Regression	0.999	0.826	0.562
Linear Regression	0.998	0.891	0.621
Support Vector Regression	0.998	0.930	0.640
MLP Regressor	0.997	1.222	0.861
POMs‐GibbsNet	**0.997**	**1.326**	**0.949**
Gradient Boosting	0.993	1.839	1.231
Extra Trees	0.992	2.086	1.126
Lasso	0.983	2.945	2.225
Random Forest	0.981	3.115	1.436
Elastic Net	0.977	3.470	2.611
Ridge	0.957	4.704	3.353

*Note:* Errors are reported in kcal mol^−1^. Bold values in Table 2 highlight our work in comparison with other classical ML protocols.

The benchmark reveals that all nonlinear regression methods achieve excellent predictive performance, with coefficients of determination exceeding 0.98 for most models. Among them, Kernel Ridge Regression provides the highest accuracy ($R^{2}=0.999$, RMSE = 0.826 kcal mol^−1^, MAE = 0.562 kcal mol^−1^), followed closely by ordinary linear regression and SVR. POMs‐GibbsNet ranks among the best‐performing models, outperforming ensemble tree‐based methods such as Gradient Boosting, Extra Trees, and Random Forest, although it does not achieve the lowest prediction error.

Interestingly, the small performance gap between the best‐performing algorithms suggests that the predictive accuracy of the workflow is determined primarily by the descriptor representation rather than by the regression model itself. Even ordinary least‐squares regression achieves an RMSE below 1 kcal mol^−1^ when trained on the complete descriptor set, indicating that the engineered graph and QTAIM descriptors capture most of the information required to predict the thermal Gibbs free‐energy correction. Nonlinear models provide additional improvements, particularly kernel‐based approaches, but these gains are comparatively modest.

These results demonstrate that the principal contribution of the present work lies in the physically informed descriptor representation and its ability to encode the structural and electronic information governing the thermal correction. POMs‐GibbsNet constitutes one effective nonlinear implementation of this descriptor framework, while the benchmark confirms that the descriptors themselves are responsible for the majority of the predictive capability.

To quantify the contribution of each descriptor family, we performed a descriptor‐group ablation study by sequentially removing graph‐topology, whole‐graph, DFT‐derived, and QTAIM descriptors while retraining the model. The complete results are reported in the Supporting Information. Briefly, graph‐topology descriptors alone already recover most of the predictive performance, whereas the addition of QTAIM descriptors provides the largest improvement. Whole‐graph descriptors produce only a modest gain, indicating that local electronic information dominates the prediction of the thermal Gibbs free‐energy correction.

To identify the descriptors driving the predictions, we performed a feature‐attribution analysis using SHapley Additive exPlanations (SHAP). The results reveal that QTAIM quantities associated with bond critical points consistently rank among the most influential variables, followed by graph‐topology descriptors. In contrast, global molecular properties such as molecular mass and charge exhibit comparatively small contributions. These observations are consistent with the descriptor‐ablation analysis and further support the conclusion that local electronic structure governs the prediction of G−E. A complete ranking of descriptor importances is provided in the Supporting Information.

### 3.3 Application in Automated Chemical Reaction Network: Reaction Gibbs Free Energies

To further assess the reliability and practical applicability of the machine learning predictions, the POMs‐GibbsNet results were validated using the POMSimulator platform. POMSimulator is capable of automatically generating extensive reaction networks for polyoxometalate systems, including thousands of possible chemical transformations derived from combinations of cluster building blocks, protonation states, and condensation pathways. This capability makes it particularly suitable for evaluating the consistency of predicted thermodynamic quantities across chemically diverse reactions. After these reaction networks are generated, POMSimulator calculates the reaction energy using the absolute Gibbs free energies of the molecules involved in the reaction. With these reaction energies, POMSimulator is able to simulate the aqueous multispecies equilibria of polyoxometalates and then compute the corresponding formation constants.

Within this validation framework, the Gibbs free energies predicted by the neural network were reconstructed by adding the predicted thermal correction term to the electronic energies obtained from the DFT calculations. In contrast, the reference values were taken directly from the Gibbs free energies computed using full DFT frequency calculations. By comparing these two quantities, it becomes possible to assess whether the machine learning model accurately reproduces the thermodynamic contributions arising from vibrational and entropic effects.

Unlike most machine‐learning models for molecular energies, which are typically validated against absolute molecular properties, we evaluate the model on the quantity ultimately required by POMSimulator: the reaction Gibbs free energy. This allows us to determine whether molecular prediction errors accumulate or cancel when propagated through the reaction network, analogous to the systematic error cancellation commonly observed in DFT calculations. To quantify this effect, we define the *cancellation factor* (CF) for each reaction as

(1) $$\text{CF}=\frac{\left|\sum_{i}\nu_{i}e_{i}\right|}{\sum_{i}|\nu_{i}e_{i}|},$$

where $e_{i}$ is the signed prediction error of species i and $\nu_{i}$ is its stoichiometric coefficient. A value of CF→0 indicates complete error cancellation, whereas CF=1 corresponds to no cancellation.

Propagating the prediction errors of the 1419 molecular species through the 10,819 reactions generated by POMSimulator yields a mean molecular absolute error of 0.98 kcal mol^−1^ (median 0.66 kcal mol^−1^) and a mean accumulated molecular error of 2.02 kcal mol^−1^ per reaction. Despite this, the mean absolute reaction error is only 1.20 kcal mol^−1^ (median 0.73 kcal mol^−1^), corresponding to a mean cancellation factor of 0.597 (median 0.605). Consequently, reaction errors are reduced by 40.3% relative to the accumulated molecular errors and by 59.8% compared with the 2.99 kcal mol^−1^ expected assuming statistically independent molecular errors. This reduction is highly significant (paired one‐sided t=−64.9, Wilcoxon $p<10^{-300}$, Cohen's $d_{z}=-0.62$, rank‐biserial correlation =−0.85). Error cancellation is widespread but not universal, with CF<0.5 for 42.8% of reactions, CF<0.25 for 24.5%, and CF<0.1 for 10.3%. Furthermore, while molecular and reaction errors remain strongly correlated (Pearson r=0.84), the substantially lower Spearman correlation (ρ=0.53) indicates that error cancellation markedly alters the ranking of the most challenging reactions.

In this validation procedure, different reaction types were considered, including condensation, acid–base, addition, and hydration reactions. These reactions represent the fundamental transformations that govern the self‐assembly and speciation of polyoxometalate in an aqueous solution. For each reaction type, reaction energies were calculated using both the DNN‐corrected Gibbs free energies and the original DFT‐derived Gibbs energies. Linear regression analyses were subsequently performed to quantify the agreement between the predicted and reference reaction energies.

Table 3 summarizes the predictive performance for each reaction class, reporting the number of reactions together with the corresponding $R^{2}$, RMSE, and MAE. Figure 4 represents the cancellation factor according to reaction type. Acid–base reactions, which account for 6944 of the 10,819 transformations, are predicted with the highest accuracy (RMSE = 1.14 kcal mol^−1^, MAE = 0.73 kcal mol^−1^, $R^{2}=0.99993$). This is particularly relevant because protonation equilibria dominate polyoxometalate speciation diagrams. In contrast, reactions involving rearrangements of the metal–oxo framework are considerably more challenging. Addition reactions (n=1668) exhibit an RMSE of 2.66 kcal mol^−1^ and an MAE of 1.96 kcal mol^−1^ ($R^{2}=0.9964$), while condensation reactions (n=2201) show the largest errors (RMSE = 4.27 kcal mol^−1^, MAE = 2.13 kcal mol^−1^, $R^{2}=0.9863$). Although only six hydration reactions are present in the dataset, they remain accurate in absolute terms (RMSE = 1.65 kcal mol^−1^) but display the weakest error cancellation (mean CF = 0.769; median CF = 1.00), suggesting that this observation should be interpreted cautiously due to the limited sample size. Interestingly, the degree of error cancellation does not directly mirror predictive accuracy: condensation reactions exhibit the lowest mean cancellation factor (CF = 0.578), indicating the strongest error cancellation, yet remain the least accurate reaction class because they also possess the largest molecular prediction errors.

**FIGURE 4:**
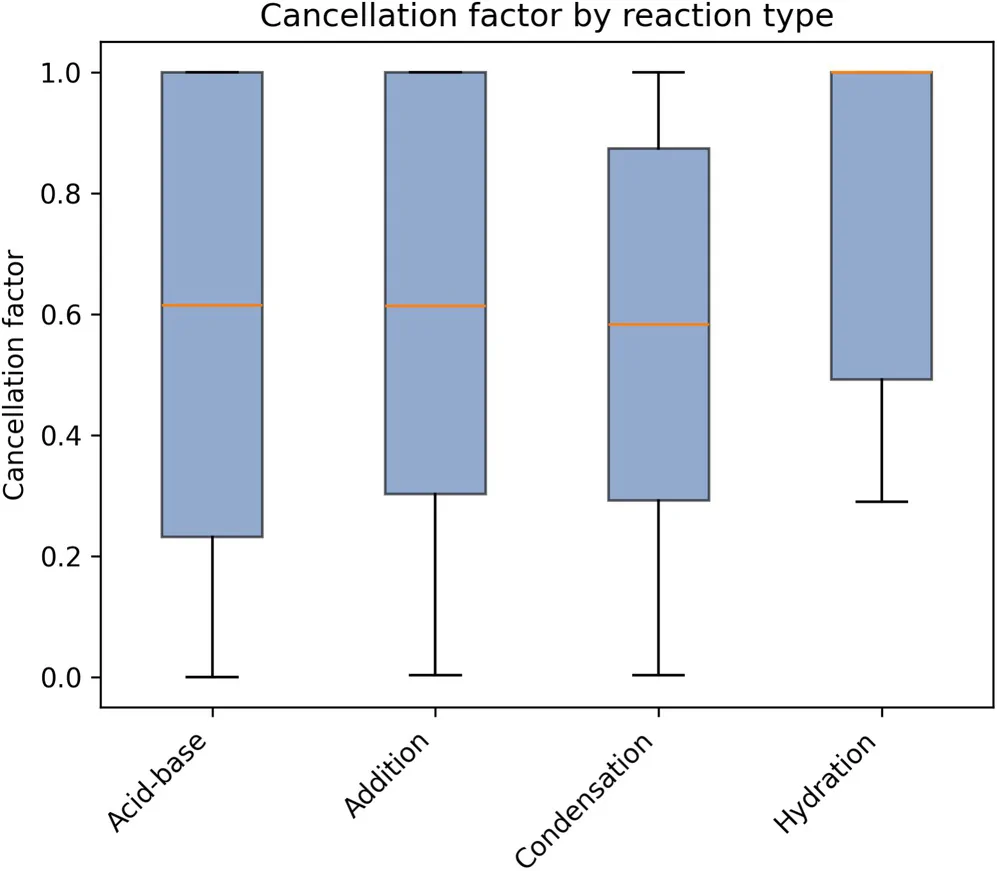
Distribution of the cancellation factor (CF) for each reaction class represented as box plots. Lower CF values indicate stronger cancellation of molecular prediction errors upon propagation to reaction Gibbs free energies, whereas CF=1 corresponds to no error cancellation. Condensation reactions exhibit the strongest error cancellation despite having the largest absolute prediction errors, while hydration reactions display the weakest cancellation.

**TABLE 3:** Statistics for the linear regressions between NN‐derived and DFT‐computed Gibbs free energies, grouped by reaction type.

Reaction type	N	$R^{2}$	RMSE	MAE
Acid–Base	6944	0.9999	1.1	0.7
Hydration	6	0.9998	2.3	1.8
Addition	1668	0.9977	2.1	1.6
Condensation	2201	0.9960	2.2	1.9

*Note:* Only the extended AABBA vector using the multiplication was considered for this study (highest $R^{2}$ and lowest RMSE and MAE), giving a total of 10,819 reactions. RMSE and MAE are in kcal/mol.

From the perspective of automated reaction‐network generation, preserving the underlying thermodynamic landscape is more important than minimizing individual numerical errors. Using POMs‐GibbsNet thermal corrections, the sign of the reaction Gibbs free energy is correctly reproduced for 97.8% of all reactions (minimum 92.5% for the Mo system), while the relative ordering of reaction energies within each chemical system is retained with a mean Spearman correlation coefficient of 0.992. Furthermore, 17 of the 19 polyoxometalate systems satisfy the predefined high‐fidelity criterion. These results demonstrate that POMs‐GibbsNet preserves the topology of the thermodynamic landscape explored by POMSimulator, with the Mo and W system representing the principal exception, as discussed in Section 4.2 of the Supporting Information.

The statistical results for each reaction category are summarized in Table 3, which reports the number of reactions analyzed together with the corresponding $R^{2}$, RMSE, and MAE values for each reaction class. In all cases, the predicted reaction energies exhibit extremely strong linear correlations with the DFT values, confirming that the machine learning corrections successfully reproduce the thermodynamic trends across the reaction network. Acid–base reactions, which represent the largest portion of the dataset, display particularly small prediction errors, with root mean squared errors close to 1 kcal· mol^−1^. Addition and condensation reactions show slightly larger deviations, although their correlations remain very strong, with $R^{2}$ values exceeding 0.99. Hydration reactions, which constitute a very small fraction of the dataset, exhibit somewhat larger uncertainties due to the limited number of available examples. Nevertheless, the overall agreement remains excellent, demonstrating that the POMs‐GibbsNet predictions are sufficiently accurate to reproduce the thermodynamic landscape generated by full DFT calculations.

To gain further insight into the behavior of the model, the reaction dataset was analyzed from two complementary perspectives: according to the reaction type and according to parent polyoxometalate system involved in the reaction. This multilevel analysis allows the identification of potential systematic trends in the predictive performance and provides insight into how structural complexity influences the thermodynamic corrections learned by the neural network.

First, the reactions were grouped according to the autocorrelation representation employed during model training. In general and in line with the results for absolute energies (Table 2), models incorporating the extended AABBA kernel produced slightly improved agreement with DFT reaction energies, particularly for transformations involving significant structural rearrangements, such as condensation and addition reactions. This behavior suggests that bond‐level descriptors and QTAIM‐derived features capture relevant electronic and topological changes occurring along these reaction pathways. Conversely, simplified atom–atom representations still produced reasonable predictions but occasionally exhibited larger deviations for reactions involving subtle bond reorganization. Notably, AA autocorrelations already provide good results for the POMSimulator framework (e.g., $R^{2}\approx 0.999$, RMSE ≈2–3 kcal mol^−1^; Table 4), helping to avoid overfitting while maintaining high predictive accuracy.

**TABLE 4:** Performance by autocorrelation representation.

Model	Operator	$R^{2}$	RMSE	MAE
AA	Sum	0.9995	2.4	1.6
AA	Division	0.9992	3.2	2.2
AA	Multiplication	0.9991	3.2	2.1
AABBA	Sum	0.9998	1.7	1.1
AABBA	Multiplication	0.9997	1.8	1.2
AABBA	Division	0.9992	3.1	2.0
Ext. AABBA	Sum	0.9998	1.6	1.0
Ext. AABBA	Multiplication	0.9998	1.6	1.1
Ext. AABBA	Division	0.9996	2.3	1.5

*Note:* Models are grouped by descriptor type. Ext. AABBA vectors consist of AABBA autocorrelation features augmented with whole‐graph properties. Each model consisted of 10,819 reactions. RMSE and MAE are reported in kcal mol^−1^.

Second, the reaction energies were analyzed by polyoxometalate system in order to determine whether predictive accuracy varies across different metal compositions and structural families. The corresponding statistical metrics are reported in Table 5. The dataset contains a wide variety of POM chemistries, including tungsten‐, molybdenum‐, niobium‐, vanadium‐, and mixed‐metal clusters. Grouping reactions according to their parent system enables identification of potential systematic biases associated with specific metal compositions or structural motifs.

**TABLE 5:** Prediction performance by POM system for the extended AABBA representation.

System	$N_{\text{reactions}}$	$R^{2}$	RMSE	MAE
AsTaW	2364	1.000	1.1	0.7
AsMoW	3532	1.000	1.0	0.8
SiTaW	19	0.999	1.3	1.0
AsW	204	0.998	1.6	1.3
PTaW	226	0.998	2.6	2.0
PMoW	1931	0.998	2.3	1.6
SiW	200	0.998	2.3	1.9
VW	252	0.997	1.8	1.4
Mo	255	0.997	2.8	2.0
PW	309	0.997	2.2	1.6
SiMo	180	0.997	2.6	2.1
Nb	118	0.996	2.5	2.0
SiMoW	38	0.995	2.1	1.3
Ta	118	0.995	2.5	2.1
AsMo	204	0.995	2.3	1.8
V	224	0.993	2.6	2.1
VMo	204	0.991	2.7	2.1
PMo	204	0.991	2.8	2.2
W	237	0.977	3.4	2.5

*Note:* RMSE and MAE are reported in kcal mol^−1^.

Grouping the reactions according to their parent polyoxometalate system reveals consistent predictive performance across the mixed‐metal families, whereas substantially larger errors are observed for several single‐metal systems. This trend mirrors the results obtained from the leave‐one‐system‐out validation (Supporting Information) and should therefore be interpreted as evidence that model performance depends on chemical similarity rather than uniform transferability across all systems.

Mixed‐metal reactions are predicted with an RMSE of 1.41 kcal mol^−1^, compared with 4.17 kcal mol^−1^ for single‐metal reactions. The most accurate predictions are obtained for the densely represented mixed‐metal systems AsMoW (RMSE = 0.84 kcal mol^−1^) and AsTaW (1.02 kcal mol^−1^), whereas the largest errors occur for the Mo system (RMSE = 11.41 kcal mol^−1^, MAE = 6.34 kcal mol^−1^, $R^{2}=0.9496$), followed by W (3.65 kcal mol^−1^), Nb (2.90 kcal mol^−1^), and Ta (2.84 kcal mol^−1^). Although the superior performance for mixed‐metal systems partly reflects their greater representation in the training set, the correlation between training‐set size and per‐system error is weak and not statistically significant (Spearman ρ=−0.205, p=0.40). This indicates that data availability alone does not explain the observed failures. Instead, the most challenging systems correspond to small single‐metal isopolyanions with highly protonated, water‐rich stoichiometries, suggesting that the underlying chemical diversity, rather than sample size, ultimately limits model transferability.

These observations highlight the importance of maintaining chemical diversity in the training dataset to ensure the transferability of the learned thermodynamic corrections.

Taken together, these analyses demonstrate that the machine learning predictions remain robust across different molecular representations, polyoxometalate systems, and reaction classes. The consistent agreement between predicted and DFT‐derived reaction energies highlights the reliability of the proposed DFT–machine learning hybrid framework. By accurately reproducing the thermodynamic corrections required to obtain Gibbs free energies, the POMs‐GibbsNet model enables rapid evaluation of extensive reaction networks while avoiding the computational cost associated with full vibrational frequency calculations. When integrated with tools such as POMSimulator, this approach provides a powerful strategy for accelerating the exploration of polyoxometalate speciation and self‐assembly pathways across large regions of chemical space.

To better understand the limitations of the proposed approach, a detailed failure analysis was performed and is presented in the Supporting Information. The analysis identifies the chemical systems and reaction classes responsible for the largest prediction errors, together with their relationship to the applicability domain and the uncertainty estimates, providing practical guidelines for the reliable application of POMs‐GibbsNet.

## 4 Data Records

### 4.1 POMs Repository: ioChem‐BD

A total of 1419 polyoxometalates, sorted into 19 different systems, were used to perform the AABBA autocorrelations, to create a fixed‐length vector that could be fed into different ML algorithms. To check whether these polyoxometalates had a heterogeneous distribution of their properties, density plots were employed for each of the graph properties (Figure SI I).

Additionally, bar plots were used to study the maximum depth of graphs. The depth can be defined as the maximum distance between two nodes of the graph. This step was important for the generation of the AA, BB, and AB autocorrelation vectors. The median value of this distribution was used to proceed with the ACs.

The resulting dataset was uploaded in the ioChem‐BD repository [68] and can be publicly accessed via the following link https://doi.org/10.19061/iochem‐bd‐1‐423.

### 4.2 Code Availability: GitLab

An open repository of the code is available in GitLab (https://gitlab.com/JBuils/poms‐gibbsnet). Additionally to the POMs‐GibbsNet model, the repository contains an adapted version of the AABBA graph kernel and an adapted version of the POMSimulator 1.0.0 release.

## 5 Conclusions

POMs‐GibbsNet provides an efficient surrogate model for predicting the thermal Gibbs free‐energy correction (G−E) of polyoxometalates, replacing computationally demanding DFT frequency calculations with a descriptor‐based machine‐learning workflow compatible with automated reaction‐network generation. Using graph‐based autocorrelation descriptors enriched with physically meaningful QTAIM features, the model accurately reproduces molecular thermal corrections while remaining fully integrated within the POMSimulator framework.

Beyond molecular accuracy, the revised validation demonstrates that the approach preserves the thermodynamic properties required for chemical‐network construction. When propagated through more than 10,000 reactions, molecular prediction errors cancel substantially, yielding reaction Gibbs free energies that closely reproduce the DFT reference values and preserve both the sign and relative ordering of reaction energies. Benchmarking against ten classical machine‐learning regressors further shows that the excellent predictive performance originates primarily from the descriptor representation rather than from the specific neural‐network architecture, highlighting the importance of physically informed descriptors for modelling complex inorganic clusters.

The additional validation performed in this work also defines the practical scope of the model. Chemically informed cross‐validation reveals excellent interpolation within the well‐represented regions of polyoxometalate chemical space while identifying reduced transferability for underrepresented single‐metal systems. Complementary uncertainty quantification and applicability‐domain analyses provide a practical mechanism for identifying predictions made outside the domain supported by the training data, thereby increasing the robustness of the workflow for automated applications.

Finally, the proposed strategy substantially reduces the computational effort associated with thermodynamic calculations while retaining the accuracy required for reaction‐network generation. Although developed for polyoxometalates, the combination of graph descriptors, QTAIM‐derived features, and uncertainty‐aware machine learning provides a general framework that can be extended to other classes of metal–oxo clusters and coordination compounds. Future work will focus on expanding the chemical diversity of the training dataset, improving transferability to underrepresented systems, and incorporating increasingly robust uncertainty calibration as larger datasets become available.

## Author Contributions

Jordi Buils adapted the AABBA graph kernel, developed and implemented the POMs‐GibbsNet neural network model, and adapted POMSimulator. Jordi Buils also optimized and tested the model's performance, generated the POMs database, and uploaded it to ioChem‐BD. Jordi Buils was the main contributor to writing the manuscript. Carles Bo and David Balcells contributed to the design and supervision of the research project, revised the manuscript, and secured funding. All authors contributed to writing and revising the manuscript.

## Funding

This work was supported by the Ministerio de Ciencia, Innovación y Universidades (Grant Nos. PID2023‐153344NB‐I00 and CEX2024‐001469‐S), Generalitat de Catalunya, RCN FRIPRO (catLEGOS project) (Grant No. 325003), RCN National Centers of Excellence (Grant No. Hylleraas Centre; 262695), and the Norwegian Supercomputing Program (NOTUR) (Grant Nos. NN4654K and NS4654K).

## Conflicts of Interest

The authors declare no conflicts of interest.

## Supporting information




**Table SI I:** Polyoxometalate families included in the dataset. **Table SI II:** Origin of the descriptor groups employed by POMs‐GibbsNet. **Table SI III:** Descriptors excluded from the feature set to prevent target leakage. **Figure SI I:** Distribution of the principal structural, electronic and thermodynamic properties across the complete dataset of 1419 polyoxometalates. The histograms illustrate the chemical diversity in terms of molecular size, composition, electronic structure and graph topology. **Figure SI II:** Predicted versus DFT‐computed thermal Gibbs free‐energy corrections for all polyoxometalate systems using the AA representation with the summation operator. **Table SI IV:** Hyperparameter search space used during the Optuna optimization of POMs‐GibbsNet. **Table SI V:** Optimal POMs‐GibbsNet hyperparameters obtained from the Optuna optimization. **Table SI VI:** Benchmark of POMs‐GibbsNet and ten classical machine‐learning regressors using identical molecular descriptors and data partitions. RMSE and MAE are reported in kcal mol^−1^. Training and prediction times correspond to the evaluated dataset. **Figure SI III:** Residual distributions and absolute prediction errors for POMs‐GibbsNet and the classical machine‐learning regressors. Residuals are defined as predicted minus DFT thermal Gibbs free‐energy corrections. **Table SI VII:** Descriptor‐group ablation for POMs‐GibbsNet. RMSE and MAE are reported in kcal mol^−1^. **Figure SI IV:** Contribution of the different descriptor groups to predictive accuracy. The contribution is expressed as the change in RMSE associated with each descriptor group; positive values indicate an increase in RMSE relative to the corresponding reference representation. **Figure SI V:** Permutation feature importance for POMs‐GibbsNet. The figure shows the 30 descriptors producing the largest increase in RMSE when individually permuted. Larger values indicate a greater dependence of the model predictions on the corresponding descriptor. **Table SI VIII:** Test‐set performance for the 19 leave‐one‐system‐out (LOSO) folds. In each fold, the complete POM system indicated in the first column was withheld from model development. RMSE and MAE are reported in kcal mol^−1^. **Table SI IX:** Test‐set performance for the leave‐one‐metal‐family‐out (LOMFO) validation. Each fold corresponds to a metal family withheld from model development. RMSE and MAE are reported in kcal mol^−1^. **Table SI X:** Benchmark of uncertainty‐quantification strategies for POMs‐GibbsNet. ECE and MCE denote expected and maximum calibration error, respectively. Coverage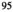 is the empirical coverage of the nominal 95% prediction interval. **Figure SI VI:** Distribution of Monte‐Carlo‐dropout predictive uncertainties across the test structures. The dashed line indicates the median uncertainty. **Table SI XI:** Applicability‐domain analysis for metal‐family holdouts. OOD denotes structures belonging to the withheld metal family, while in‐domain values correspond to structures represented by related metal families in the training distribution. Uncertainty and error ratios are calculated as OOD divided by in‐domain values. **Figure SI VII:** Distribution of Monte‐Carlo‐dropout predictive uncertainty across POM families. Triangles indicate the mean uncertainty and circles denote individual high‐uncertainty observations. **Table SI XII:** Predictive uncertainty and absolute error by POM system. N is the number of test structures, $\sigma_{\text{unc}}$ is the standard deviation of the Monte‐Carlo‐dropout predictive distribution, and MAE is the absolute prediction error in kcal mol^−1^. The fraction gives the proportion of the complete dataset represented by each system. **Table SI XIII:** Composition of the reaction network generated by POMSimulator for the complete dataset. The network comprises 1618 molecular species connected through 10,819 chemically balanced reactions classified according to their transformation type. **Figure SI VIII:** Distribution of signed reaction free‐energy prediction errors, $\Delta G^{\text{pred}}-\Delta G^{\text{DFT}}$, computed over the complete reaction network. The vertical red line indicates the mean signed error, while the dashed green line denotes zero prediction error. **Figure SI IX:** Distribution of absolute reaction free‐energy prediction errors for the four reaction classes identified by POMSimulator: acid–base, addition, condensation, and hydration. Boxes represent the interquartile range, the horizontal line indicates the median, and whiskers extend to 1.5 times the interquartile range. **Table SI XIV:** Prediction statistics for reaction Gibbs free energies computed over the complete POMSimulator reaction network. Molecular prediction errors are propagated through the reaction stoichiometry and compared with DFT reference reaction energies. **Table SI XV:** Summary statistics of the reaction error‐cancellation analysis. The cancellation factor is defined as $\left|\Delta G_{\text{err}}|/\sum_{i}|e_{i}\right|$, where values below one indicate partial cancellation of molecular prediction errors. **Figure SI X:** Relationship between the accumulated molecular prediction error, $\sum_{i}|e_{i}|$, and the corresponding reaction free‐energy prediction error, $\left|\Delta G^{\text{pred}}-\Delta G^{\text{DFT}}\right|$, for all reactions generated by POMSimulator. The dashed line represents the limiting case in which molecular errors accumulate without cancellation (y=x). Pearson and Spearman correlation coefficients between both quantities are reported in the inset. **Figure SI XI:** Distribution of signed reaction free‐energy prediction errors grouped according to the POM family associated with each reaction. Boxes represent the interquartile range, the horizontal line indicates the median, and whiskers extend to 1.5 times the interquartile range. **Table SI XVI:** Reaction prediction errors grouped by reaction class. Median absolute reaction errors and cancellation factors were computed for every reaction type in the complete POMSimulator reaction network. **Figure SI XII:** Distribution of the cancellation factor, computed for all reactions in the network. The solid red line marks the median cancellation factor, whereas the dashed gray line corresponds to CF=1, indicating the absence of error cancellation. **Figure SI XIII:** Distribution of the cancellation factor grouped by reaction class. The cancellation factor is defined as $\left|\Delta G_{\text{err}}|/\sum_{i}|e_{i}\right|$, where values approaching unity indicate little or no cancellation of molecular prediction errors. **Table SI XVII:** Wall‐clock computational cost of the GibbsNet workflow relative to a conventional DFT vibrational frequency calculation. The reported speedup is computed with respect to the DFT frequency calculation, which represents the computational bottleneck replaced by POMs‐GibbsNet.

## Data Availability

The data that support the findings of this study are openly available in POMs‐GibbsNet at https://gitlab.com/JBuils/poms‐gibbsnet.
