# Supplementary material for: POMs‐GibbsNet: A Deep Neural Network for the Prediction of Thermodynamic Parameters of Polyoxometalates
Source: J Comput Chem. 2026 Oct 21;47(28):e70510. doi: 10.1002/jcc.70510 (PMC13685485; doi:10.1002/jcc.70510)
Supplement: Supplementary file 1 [file JCC-47-0-s001.pdf]

Supporting Information for: POMs-GibbsNet: a Deep Neural Network for the prediction of thermodynamic parameters of polyoxometalates

Jordi Buils[a,*], Carles Bo[a,b], and David Balcells[c,*]

[a]Institute of Chemical Research of Catalonia (ICIQ), The Barcelona Institute of Science and Technology (BIST), Av. Països Catalans, 16, 43007 Tarragona, Spain.
[b]Departament de Química Física i Inorgànica, Universitat Rovira i Virgili, Marcel·li Domingo s/n, 43007, Tarragona, Spain.
[c]Hylleras Centre for Quantum Molecular Sciences, Department of Chemistry, University of Oslo, 0315 Oslo, Norway
[*]Corresponding author

September 12, 2026

Contents

1 Dataset and descriptor overview **3**
- 1.1 Dataset composition . . . 3
- 1.2 Descriptor overview . . . 3
- 1.3 Descriptor provenance and target independence . . . 3

2 Molecular representations **6**
- 2.1 AA descriptors . . . 6
- 2.2 AABBA descriptors . . . 7
- 2.3 Extended AABBA descriptors . . . 7
- 2.4 Comparison of molecular representations . . . 7

3 POMs-Gibbsnet and model development **9**
- 3.1 POMs-GibbsNet architecture . . . 9
- 3.2 Hyperparameter optimization . . . 9
- 3.3 Benchmark against classical machine-learning regressors . . . 10
- 3.4 Descriptor ablation and feature attribution . . . 11
  - 3.4.1 Descriptor-group ablation . . . 11
  - 3.4.2 Permutation feature importance . . . 13

4 Generalization and validation **16**
- 4.1 Random split . . . 16
- 4.2 Leave-one-system-out cross-validation . . . 16
- 4.3 Leave-one-metal-family-out cross-validation . . . 17

5 Uncertainty quantification and applicability domain **18**
- 5.1 Monte-Carlo dropout uncertainty . . . 19
- 5.2 Deep-ensemble uncertainty . . . 19
- 5.3 Applicability-domain analysis . . . 20
- 5.4 Uncertainty across chemical space . . . 21

6 Error analysis and limitations **25**
- 6.1 Error by chemical system . . . 25
- 6.2 Failure modes and difficult structures . . . 25
- 6.3 Chemical characteristics associated with prediction error . . . 26
- 6.4 Limitations and applicability . . . 26

7 POMSimulator validation **27**
- 7.1 Reaction-network construction . . . 27
- 7.2 Reaction-level validation methodology . . . 27
- 7.3 Error cancellation analysis . . . 28
- 7.4 Reaction-class performance . . . 30

8 Computational efficiency **34**
- 8.1 Computational cost model . . . 34
- 8.2 Scaling analysis . . . 35

1 Dataset and descriptor overview

1.1 Dataset composition

The dataset used to train and evaluate POMs-GibbsNet consists of 1419 optimized polyoxometalate (POM) structures spanning 19 chemically distinct systems:

Table SI I: Polyoxometalate families included in the dataset.

AsMo	AsMoW	AsTaW	AsW	Mo
Nb	PMo	PMoW	PTaW	PW
SiMo	SiMoW	SiTaW	SiW	Ta
V	VMo	VW	W	

Each entry corresponds to a unique optimized molecular structure, including different protonation states, nuclearities and metal compositions. Together, these systems cover a broad range of molecular sizes, charges and chemical environments representative of the polyoxometalate families considered in this work.

To characterize the diversity of the dataset, several global molecular properties were extracted for every structure, including the number of atoms, number of bonds, number of electrons, total molecular charge, molecular mass, HOMO and LUMO energies, HOMO–LUMO gap, electronic energy and thermal correction to the Gibbs free energy ($G - E$). In addition, the graph depth, defined as the longest shortest-path distance between any two nodes in the molecular graph, was computed as a simple topological descriptor of cluster size and connectivity.

Figure SI I summarizes the distribution of these molecular and electronic properties across the complete dataset. The wide range of values illustrates the structural and electronic diversity of the considered POM families, providing a suitable foundation for training and validating machine-learning models capable of interpolating across chemically related systems.

1.2 Descriptor overview

The descriptors employed throughout this work combine information obtained at different stages of the electronic-structure workflow. Structural information is extracted directly from the optimized molecular geometry, electronic descriptors are obtained from a single-point DFT calculation, and local bonding information is incorporated through the quantum theory of atoms in molecules (QTAIM). No descriptor requires a vibrational frequency calculation, ensuring that the proposed workflow remains independent of the computationally demanding Hessian evaluation that POMs-GibbsNet is designed to replace.

For clarity, the complete descriptor set is divided into four groups: graph-topological descriptors, electronic descriptors, QTAIM-derived descriptors, and whole-graph physicochemical properties. Their mathematical construction is described in Section S2, while Table S1 summarizes the provenance of every descriptor and the computational stage at which it is generated.

1.3 Descriptor provenance and target independence

The principal objective of POMs-GibbsNet is to replace the computationally expensive DFT vibrational analysis required to evaluate the thermal Gibbs free-energy correction ($G - E$). Consequently, all input descriptors were selected such that they can be obtained before performing a frequency calculation. Structural information is extracted from the optimized molecular geometry, electronic descriptors are computed from a single-point DFT calculation, and

local bonding information is obtained from a subsequent QTAIM analysis. No vibrational frequencies, thermal corrections or thermodynamic quantities are used as model inputs.

Table SI II summarizes the provenance of every descriptor group and the computational stage at which it is generated. Approximately one third of the descriptor set originates from the optimized molecular geometry, one third from QTAIM properties, one fifth from single-point DFT calculations, and the remaining descriptors correspond to whole-graph identity channels used during the graph construction.

Table SI II: Origin of the descriptor groups employed by POMs-GibbsNet.

Producing stage	Features	Fraction	Requires frequency calculation?
Geometry optimisation	Z, T, S, χ, bond-order	36 (33%)	No
Single-point DFT	Q_m, Q_d, Q_q	24 (22%)	No
QTAIM	d, M, ε, ρ, $\nabla^2\rho$, $\Diamond$, G_b, V_b, H_b	36 (33%)	No
Dataset metadata	Identity channel (I)	12 (11%)	No
Frequency calculation	None	0 (0%)	—

To further demonstrate the absence of information leakage, all descriptors directly related to the target property were excluded from the final feature set. In particular, quantities that require vibrational analysis, including the Gibbs free energy, enthalpy, entropy and vibrational frequencies, were removed before model training. Although the electronic energy is used to define the target quantity ($G - E$), it is likewise excluded from the descriptors to ensure that the network predicts the complete thermal correction independently of any information defining the regression target (SI III.

Table SI III: Descriptors excluded from the feature set to prevent target leakage.

Descriptor	Reason for exclusion	Producing stage
`gibbs_corr`	Regression target ($G - E$)	Frequency calculation
`gibbs`	Defines the target quantity	Frequency calculation
`electronic`	Defines the target quantity	Single-point DFT
`enthalpy`	Thermal correction	Frequency calculation
`entropy`	Thermal correction	Frequency calculation
`high_freq`, `low_freq`	Vibrational frequencies	Frequency calculation

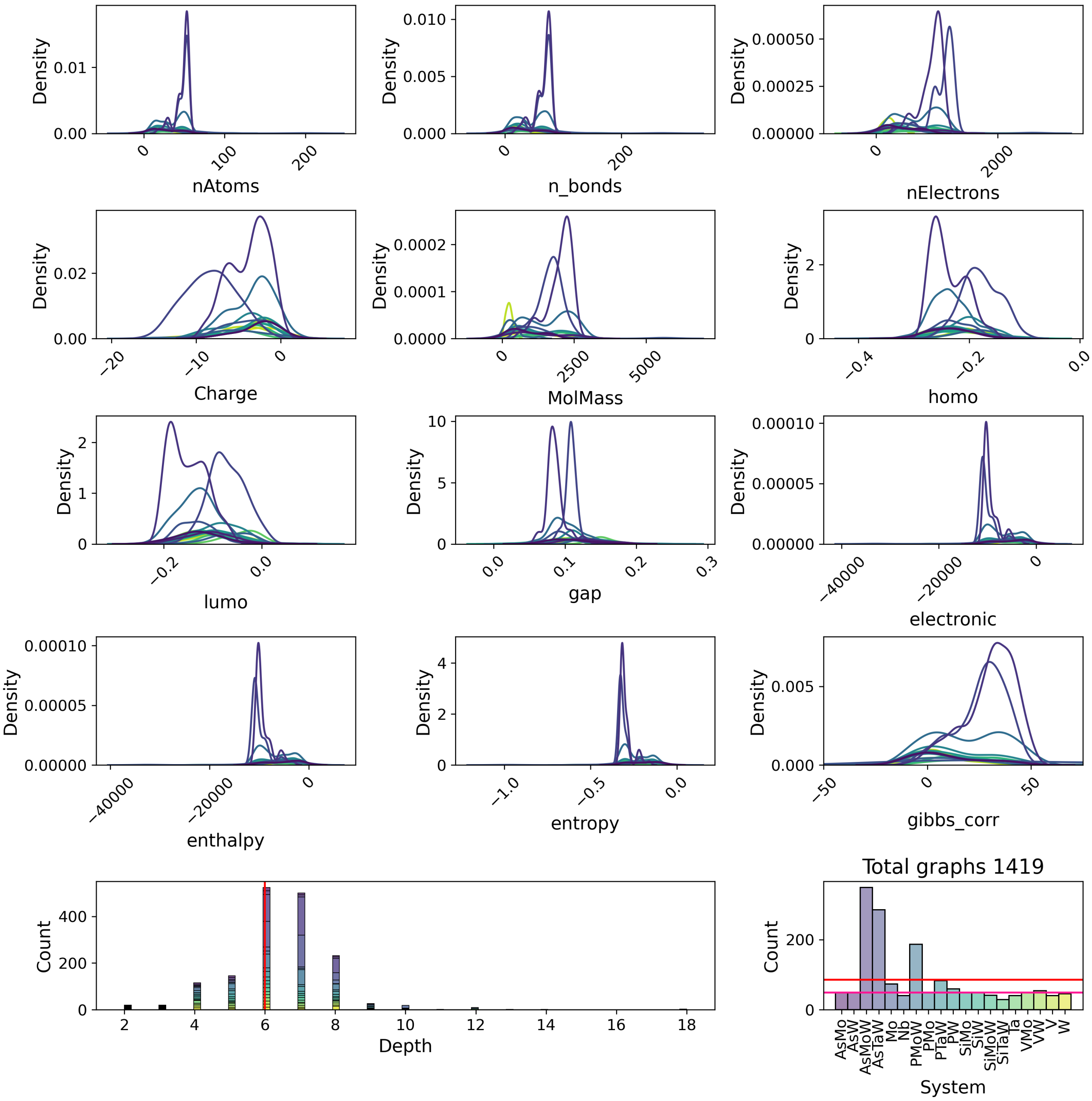

Figure SI I: Distribution of the principal structural, electronic and thermodynamic properties across the complete dataset of 1419 polyoxometalates. The histograms illustrate the chemical diversity in terms of molecular size, composition, electronic structure and graph topology.

2 Molecular representations

The prediction of the thermal Gibbs free-energy correction requires a molecular representation that captures both the connectivity of the polyoxometalate framework and its local chemical environment. Three related autocorrelation-based representations were investigated in this work: the atom–atom (AA), atom–atom–bond–bond–atom (AABBA), and extended AABBA representations. These representations progressively incorporate information about molecular topology, atomic properties, bond properties, and global molecular characteristics.

All three representations are constructed from the molecular graph obtained after geometry optimisation. The graph consists of atoms as nodes and chemical bonds as edges, with atomic and bond properties assigned to the corresponding graph elements. Autocorrelation functions are then used to propagate these properties through the graph and generate descriptors that encode both local chemical environments and longer-range topological relationships.

The three representations differ primarily in the type and amount of information included. The AA representation contains atom-centred autocorrelation descriptors and therefore provides a relatively compact description of the molecular topology and atomic environments. The AABBA representation extends this description by incorporating bond properties, allowing the model to distinguish between molecular environments that may have similar atomic connectivity but different bonding characteristics. Finally, the extended AABBA representation augments the graph-based descriptors with whole-graph molecular and electronic properties, providing a global description of the POM in addition to the local and topological information encoded by the autocorrelation descriptors.

The resulting representations were evaluated using the same modelling framework and data partitions, allowing the effect of the descriptor representation to be assessed independently of the training procedure. A comparison of their predictive performance is provided in Section **??** and in the main text.

2.1 AA descriptors

The atom–atom (AA) representation describes the molecular graph using atom-centred properties and their autocorrelations along the graph. For a molecular graph $G = (V, E)$, each atom $i \in V$ is assigned a set of atomic properties. The autocorrelation descriptors quantify the relationship between pairs of atoms separated by a given topological distance.

For an atomic property P, the autocorrelation function at topological distance d is defined as

$$AC_d(P) = \sum_{i,j} P_i P_j \, \delta(d_{ij}, d), \tag{1}$$

where P_i and P_j are the values of the selected atomic property for atoms i and j, respectively, d_{ij} is the shortest-path distance between the two atoms in the molecular graph, and δ is the Kronecker delta. The resulting descriptors therefore encode correlations between atomic properties as a function of graph distance.

The AA representation used in this work includes the atomic properties defined from the optimized molecular structures and the corresponding electronic-structure calculations. These descriptors provide a compact representation of the POM topology while retaining information about the chemical nature of the atoms. Because the autocorrelation is evaluated over increasing graph distances, the representation captures both local environments and longer-range structural relationships within the cluster.

The AA representation constitutes the simplest of the three representations considered here and serves as a reference for assessing the additional information provided by bond-centred

descriptors and whole-graph properties.

2.2 AABBA descriptors

The atom–atom bond–bond and atom–bond (AABBA) representation extends the AA representation by incorporating properties associated with chemical bonds. Rather than describing a molecular environment exclusively through correlations between atomic properties, AABBA descriptors encode sequences involving two atoms and the bonds connecting them. This allows the representation to incorporate information about both atomic identity and the local bonding environment.

For two atoms connected through a sequence of bonds, the AABBA descriptors combine atomic and bond properties according to the corresponding graph path. In this way, the representation distinguishes molecular environments that may exhibit similar atomic connectivity but differ in bond characteristics. The resulting descriptors retain the distance-dependent nature of the AA autocorrelations while providing additional sensitivity to the connectivity and chemical environment of the metal–oxo framework.

The AABBA representation was evaluated using the same autocorrelation operators and modelling procedure as the AA representation. This enables a direct assessment of whether the inclusion of bond information provides a measurable improvement in the prediction of $G - E$.

2.3 Extended AABBA descriptors

The extended AABBA representation combines the local and topological information of the AABBA descriptors with a set of whole-graph molecular and electronic properties. The motivation for this extension is that autocorrelation descriptors describe relationships within the molecular graph, whereas some thermodynamic properties may also depend on global characteristics of the entire POM.

The extended representation contains the AABBA autocorrelation descriptors together with seven whole-graph properties: the number of atoms, number of electrons, molecular charge, molecular mass, HOMO energy, LUMO energy, and HOMO–LUMO gap. The first four quantities are obtained directly from the optimized molecular structure, while the frontier-orbital quantities are obtained from the corresponding single-point DFT calculation. None of these descriptors requires a vibrational frequency calculation.

The complete extended representation contains 115 features. The whole-graph descriptors therefore provide global molecular information while the autocorrelation descriptors retain information about local chemical environments and graph topology. The resulting representation combines three complementary levels of information: atomic properties, bond and graph connectivity, and global molecular and electronic properties.

The three representations were compared using identical modelling protocols. Although the extended AABBA representation provides the largest feature set, the comparison shows that the simpler AA and AABBA representations already provide high predictive accuracy. This observation motivated the subsequent descriptor-ablation and feature-attribution analyses, which are presented in Section 3.4.

2.4 Comparison of molecular representations

To assess the influence of descriptor complexity on predictive performance, the three molecular representations described above were evaluated using the same neural-network architecture and data partitioning. Particular attention was given to the two representations defining the

extremes of the descriptor space explored in this work: the AA representation with the summation operator and the extended AABBA representation with the product operator. These representations contain 33 and 126 features, respectively, and therefore differ substantially in the amount and type of chemical information provided to the model.

The predictive performance of the two representations is illustrated in Figures **??** and SI II, which show the predicted versus DFT-computed thermal Gibbs free-energy corrections for all POM systems. The extended AABBA representation exhibits a close correlation with the reference values, achieving $R^2 = 0.997$, RMSE $= 1.3$ kcal mol^{-1}, and MAE $= 0.8$ kcal mol^{-1}. The inclusion of bond-level and whole-graph descriptors therefore provides additional information that can improve the prediction of the thermal correction.

The simpler AA representation also provides a high level of predictive accuracy, despite containing substantially fewer features. The AA model achieves $R^2 = 0.993$, RMSE $= 2.1$ kcal mol^{-1}, and MAE $= 1.5$ kcal mol^{-1}, with predictions remaining closely distributed around the diagonal (Figure SI II). Thus, removing bond-level and whole-graph information results in only a moderate degradation in predictive performance.

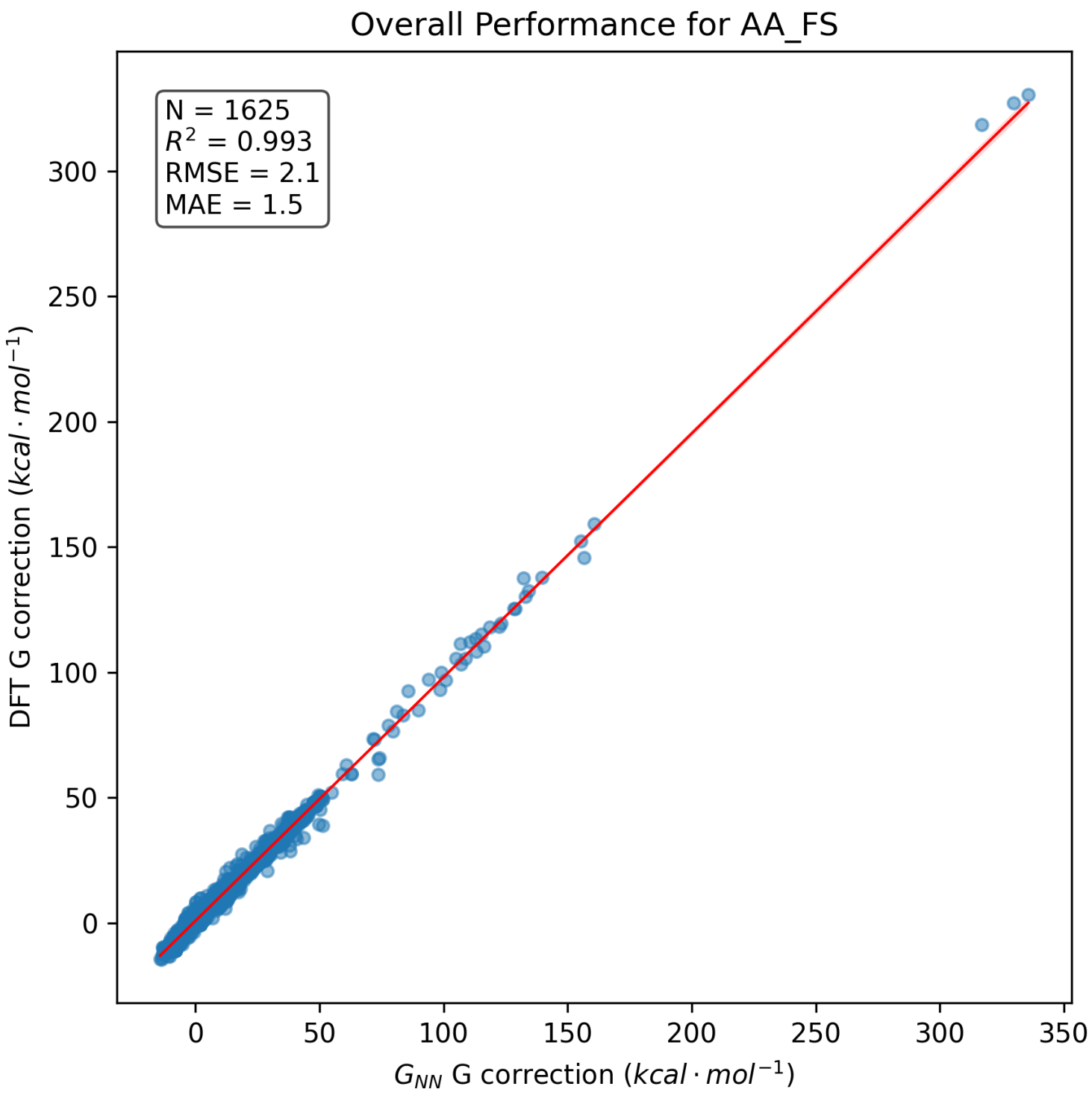

Figure SI II: Predicted versus DFT-computed thermal Gibbs free-energy corrections for all polyoxometalate systems using the AA representation with the summation operator.

A comparison of all representation and aggregation-operator combinations is provided in Table **??**. All configurations achieve high predictive accuracy under the random data partition, with $R^2 \geq 0.985$ and MAE ≤ 2.2 kcal mol^{-1}. The relatively small difference between the simplest and most extended representations demonstrates that a substantial fraction of the information required to predict $G - E$ is already encoded in the molecular topology and atomic properties captured by the AA representation.

These results indicate that increasing descriptor dimensionality does not lead to a proportional improvement in predictive performance. Although the extended AABBA representation provides the lowest errors among the representations considered, the simpler AA representation achieves comparable accuracy with approximately one quarter of the number of features. This observation motivated the subsequent descriptor-group ablation and feature-attribution

analyses, which were used to identify which components of the representation contribute most strongly to the prediction (Section 3.4).

3 POMs-Gibbsnet and model development

3.1 POMs-GibbsNet architecture

POMs-GibbsNet was implemented as a fully connected feed-forward neural network designed to map the molecular descriptor vector onto the thermal Gibbs free-energy correction, $G - E$. The final architecture was selected through hyperparameter optimisation using Optuna. The optimised network contains five hidden layers, with the first hidden layer containing 105 neurons. The hidden-layer dimensionality is progressively reduced towards the single-neuron output layer, which provides the predicted value of $G - E$.

The network uses the exponential linear unit (ELU) activation function in the hidden layers. Batch normalization was not used in the final architecture. The optimised learning rate was 1.4214×10^{-3} and the batch size was 18. The best validation objective obtained during the hyperparameter optimisation was $R^2 = 0.99818$.

The input dimensionality depends on the molecular representation employed. The AA, AABBA, and extended AABBA representations were therefore evaluated using the same network architecture and training procedure, with only the input feature vector changing between representations. The output layer contains a single neuron corresponding to the predicted thermal Gibbs free-energy correction, $G - E$.

3.2 Hyperparameter optimization

The hyperparameters of POMs-GibbsNet were optimized using the Optuna framework. A total of 50 trials were performed, with the coefficient of determination (R^2) on the validation set used as the optimization objective. The optimization was configured to maximize R^2, while the independent test set was excluded from the optimization procedure and reserved exclusively for the final model evaluation.

The Optuna search space included the initial hidden-layer dimensionality, number of hidden layers, activation function, batch normalization, learning rate, and batch size. The hidden dimension was varied from 32 to 128 neurons in increments of one, while the number of hidden layers was varied from 2 to 12. Three activation functions were considered: ELU, ReLU, and LeakyReLU. Batch normalization was treated as a categorical parameter and could either be enabled or disabled. The learning rate was sampled on a logarithmic scale between 10^{-4} and 10^{-2}, and the batch size was varied from 16 to 128 in increments of two. The complete search space is summarized in Table SI IV.

A median-based pruning strategy was enabled during the optimization to terminate poorly performing trials before completion. One startup trial was allowed before pruning was activated, with pruning evaluations performed every five optimization steps after a five-step warm-up period. Each training trial was allowed to run for a maximum of 100 epochs.

The optimal configuration consisted of five hidden layers with an initial hidden dimension of 105 neurons, using the ELU activation function without batch normalization. The optimized learning rate was 1.4214×10^{-3} and the batch size was 18. The best validation objective obtained during the optimization was $R^2 = 0.99818$. The resulting hyperparameter configuration was subsequently fixed for the final model evaluation.

Table SI IV: Hyperparameter search space used during the Optuna optimization of POMs-GibbsNet.

Hyperparameter	Search space	Sampling
Initial hidden dimension	32–128	Integer, step = 1
Number of hidden layers	2–12	Integer, step = 1
Activation function	ELU, ReLU, LeakyReLU	Categorical
Batch normalization	True, False	Categorical
Learning rate	10^{-4}–10^{-2}	Log-uniform
Batch size	16–128	Integer, step = 2

Table SI V: Optimal POMs-GibbsNet hyperparameters obtained from the Optuna optimization.

Hyperparameter	Optimal value
Number of hidden layers	5
Initial hidden dimension	105
Activation function	ELU
Batch normalization	No
Learning rate	1.4214×10^{-3}
Batch size	18

3.3 Benchmark against classical machine-learning regressors

To determine whether the predictive performance of POMs-GibbsNet originates primarily from the neural-network architecture or from the molecular descriptor representation, the optimized neural network was benchmarked against a set of classical machine-learning regression algorithms. All models were trained and evaluated using the same molecular descriptors, target variable, and data partitioning, ensuring that differences in performance could be attributed to the regression algorithm rather than to differences in the input representation or test set.

The benchmark included ten classical regression models: kernel ridge regression, ordinary linear regression, support vector regression (SVR), multilayer perceptron regression (MLP), gradient boosting regression, extra trees regression, Lasso regression, random forest regression, elastic net regression, and ridge regression. POMs-GibbsNet was included as the reference neural-network model. The models were evaluated using the coefficient of determination (R^2), root-mean-square error (RMSE), and mean absolute error (MAE), with the latter two metrics reported in kcal mol^{-1}. Pearson and Spearman correlation coefficients were also calculated to characterize the agreement and preservation of the ordering of the DFT reference values.

The complete benchmark results are reported in Table SI VI. Kernel ridge regression provides the best overall performance, with $R^2 = 0.9987$, RMSE = 0.826 kcal mol^{-1}, and MAE = 0.562 kcal mol^{-1}. Ordinary linear regression and SVR also outperform POMs-GibbsNet, with RMSE values of 0.891 and 0.930 kcal mol^{-1}, respectively. The scikit-learn MLP regressor achieves a comparable performance to POMs-GibbsNet, whereas the tree-based and sparse linear models generally exhibit larger errors.

POMs-GibbsNet achieves $R^2 = 0.9966$, RMSE = 1.326 kcal mol^{-1}, and MAE = 0.949 kcal mol^{-1}. These results demonstrate that the neural network is not the most accurate estimator for the present interpolation task. Instead, several substantially simpler regression algorithms achieve equal or better accuracy when provided with the same descriptor representation. This comparison therefore indicates that a significant part of the predictive capability of the workflow

Table SI VI: Benchmark of POMs-GibbsNet and ten classical machine-learning regressors using identical molecular descriptors and data partitions. RMSE and MAE are reported in kcal mol^{-1}. Training and prediction times correspond to the evaluated dataset.

Model	R^2	RMSE	MAE	Pears	Spear	Train t (s)	Pred t (s)	Params
Kernel Ridge	0.9987	0.826	0.562	0.9994	0.9985	0.0808	0.0090	–
Linear Regression	0.9985	0.891	0.621	0.9993	0.9987	0.0172	0.0002	109
SVR	0.9983	0.930	0.640	0.9992	0.9982	0.0893	0.0097	–
MLP Regressor	0.9971	1.222	0.861	0.9988	0.9969	0.4033	0.0004	7041
POMs-GibbsNet	0.9966	1.326	0.949	0.9984	0.9963	2.6306	0.0034	31291
Gradient Boosting	0.9934	1.839	1.231	0.9971	0.9934	4.2212	0.0011	–
Extra Trees	0.9915	2.086	1.126	0.9960	0.9945	0.3325	0.0332	–
Lasso	0.9831	2.945	2.225	0.9920	0.9789	0.0055	0.0003	109
Random Forest	0.9811	3.115	1.436	0.9916	0.9915	0.2455	0.0225	–
Elastic Net	0.9765	3.470	2.611	0.9892	0.9750	0.0051	0.0003	109
Ridge	0.9569	4.704	3.353	0.9808	0.9682	0.0017	0.0003	109

originates from the molecular representation itself rather than from the depth of the neural-network architecture.

The residual distributions provide additional information beyond the global performance metrics (Figure SI III). Kernel ridge regression, linear regression, and SVR exhibit the narrowest residual distributions, with most predictions concentrated close to zero. POMs-GibbsNet also produces a relatively compact distribution centered around zero, although its tails are broader than those of the three best-performing models. The tree-based and regularized linear models show progressively broader absolute-residual distributions and more pronounced tails, indicating a greater frequency of large prediction errors. These distributions are consistent with the differences observed in RMSE and MAE and illustrate that the poorer performance of several models is associated not only with a broader central error distribution but also with increased sensitivity to larger deviations.

The benchmark is intended as a comparison of predictive performance under the same data conditions rather than as an exhaustive hyperparameter optimization of every classical algorithm. The detailed benchmark values, correlation coefficients, computational times, and parameter counts are reported in Table SI VI.

3.4 Descriptor ablation and feature attribution

To determine which components of the molecular representation are responsible for the predictive performance of POMs-GibbsNet, two complementary analyses were performed. First, a systematic descriptor-group ablation was used to quantify the effect of removing or combining groups of descriptors. Second, permutation feature importance was used to identify the individual descriptors that contribute most strongly to the predictions. Together, these analyses distinguish the contribution of broad classes of chemical information from the importance of individual features.

3.4.1 Descriptor-group ablation

The complete representation contains 115 descriptors divided into topology, bond, QTAIM, electronic, and global molecular descriptor groups. Models were trained using different combinations of these groups while keeping the neural-network architecture, training procedure,

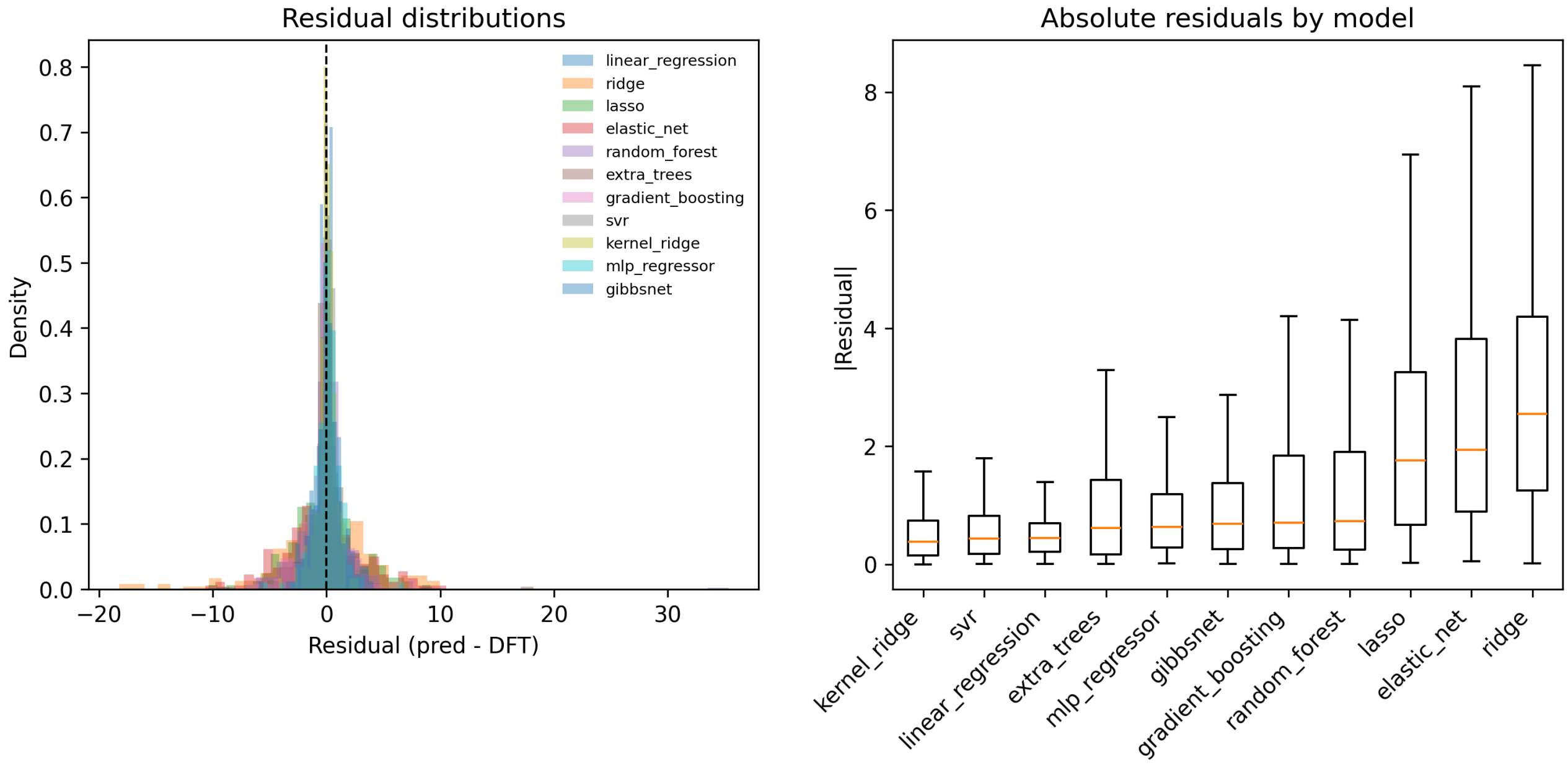

Figure SI III: Residual distributions and absolute prediction errors for POMs-GibbsNet and the classical machine-learning regressors. Residuals are defined as predicted minus DFT thermal Gibbs free-energy corrections.

and evaluation data unchanged. The complete descriptor set provides $R^2 = 0.9971$, RMSE $= 1.21$ kcal mol^{-1}, and MAE $= 0.89$ kcal mol^{-1}.

Remarkably, graph topology alone, represented by only 20 descriptors, already provides strong predictive performance, with $R^2 = 0.9915$, RMSE $= 2.09$ kcal mol^{-1}, and MAE $= 1.34$ kcal mol^{-1}. Thus, the topology-only representation recovers approximately 99.4% of the R^2 obtained with the complete representation. This result demonstrates that the molecular connectivity encoded by the graph contains a substantial fraction of the information required to predict the thermal Gibbs free-energy correction.

The QTAIM descriptor group provides the largest improvement when combined with topology. The topology + QTAIM representation contains 52 descriptors and reaches $R^2 = 0.9952$, RMSE $= 1.57$ kcal mol^{-1}, and MAE $= 1.17$ kcal mol^{-1}. The importance of QTAIM information is further supported by the leave-one-group-out experiments. Removing QTAIM descriptors from the complete representation decreases the performance from $R^2 = 0.9971$ to 0.9962, whereas removal of the electronic and global descriptor groups results in smaller changes, giving $R^2 = 0.9968$ and 0.9970, respectively.

The analysis also demonstrates that increasing descriptor dimensionality does not necessarily improve predictive performance. In particular, adding the bond descriptor group to topology alone decreases the performance to $R^2 = 0.9841$, despite increasing the number of descriptors from 20 to 52. In contrast, the topology + bond + QTAIM representation reaches $R^2 = 0.9956$ and RMSE $= 1.51$ kcal mol^{-1}. The effect of a descriptor group is therefore dependent on the other information already present in the representation, highlighting the importance of descriptor interactions and correlations.

Overall, the ablation analysis identifies graph topology as the principal baseline source of predictive information and QTAIM descriptors as the most important complementary descriptor group. Electronic and global molecular descriptors provide smaller additional improvements, while the contribution of bond descriptors is strongly representation-dependent. The complete ablation results are reported in Table SI VII.

The ablation analysis also demonstrates that increasing the number of descriptors does not

Table SI VII: Descriptor-group ablation for POMs-GibbsNet. RMSE and MAE are reported in kcal mol^{-1}.

Key	Representation	Features	R^2	RMSE	MAE
A	Topology	20	0.9915	2.091	1.342
B	Topology + Bond	52	0.9841	2.857	2.019
C	Topology + QTAIM	52	0.9952	1.574	1.173
D	Topology + Electronic	47	0.9929	1.912	1.309
E	Topology + Global	24	0.9943	1.715	1.154
F	Topology + Bond + QTAIM	84	0.9956	1.511	1.132
G	Topology + Bond + Electronic	79	0.9947	1.643	1.113
H	Topology + Bond + Global	56	0.9943	1.703	1.125
I	All except QTAIM	83	0.9962	1.391	0.919
J	All except Electronic	88	0.9968	1.291	0.917
K	All except Global	111	0.9970	1.243	0.883
L	Full descriptor set	115	0.9971	1.214	0.893

necessarily improve predictive performance. In particular, adding the bond descriptor group to topology alone reduces the performance to $R^2 = 0.9841$, despite increasing the number of features from 20 to 52. When combined with QTAIM descriptors, however, the bond descriptors provide a modest additional improvement, with the topology + bond + QTAIM representation reaching $R^2 = 0.9956$ and RMSE = 1.51 kcal mol^{-1}. This indicates that the usefulness of a descriptor group depends on the complementary information already present in the representation and that descriptor dimensionality alone is not a reliable predictor of model performance.

The complete results of the descriptor-group combinations and leave-one-group-out experiments are summarized in Table SI VII. Overall, the analysis demonstrates that graph topology provides a strong baseline representation, while QTAIM descriptors supply the most important additional information. Electronic and global descriptors contribute comparatively smaller improvements once topology and QTAIM information are available.

3.4.2 Permutation feature importance

Descriptor-group ablation was complemented by a permutation feature-importance analysis to determine which individual descriptors contribute most strongly to the predictive performance of POMs-GibbsNet. For each descriptor, its values were randomly permuted while the remaining features were left unchanged, and the resulting increase in prediction error was used as the importance measure. A larger increase in RMSE therefore indicates that the model relies more strongly on the corresponding descriptor for its predictions.

Figure SI V shows the 30 descriptors with the largest permutation importance. The most influential features include several QTAIM descriptors associated with local electronic and bonding properties, such as multipole-related quantities, electron-density descriptors, and bond-order descriptors. Several autocorrelation descriptors also appear prominently in the ranking, demonstrating that both local chemical information and graph-based structural information contribute to the prediction. The number of atoms (n_{Atoms}) is also among the most influential global descriptors.

The feature-level analysis is consistent with the descriptor-group ablation. QTAIM descriptors are strongly represented among the highest-ranked individual features, supporting the conclusion that the QTAIM block provides important complementary information beyond

graph topology. At the same time, the presence of numerous autocorrelation descriptors among the most important features confirms that the high performance of the model is not dependent exclusively on electronic descriptors, but emerges from the combination of topological and chemically informed information.

Permutation importance should be interpreted as a measure of predictive dependence rather than as a causal measure of descriptor importance. In particular, correlated descriptors can partially substitute for one another, such that permuting one descriptor may underestimate its importance if related information remains available elsewhere in the feature vector. The feature ranking is therefore considered together with the descriptor-group ablation rather than as an independent determination of causal relevance.

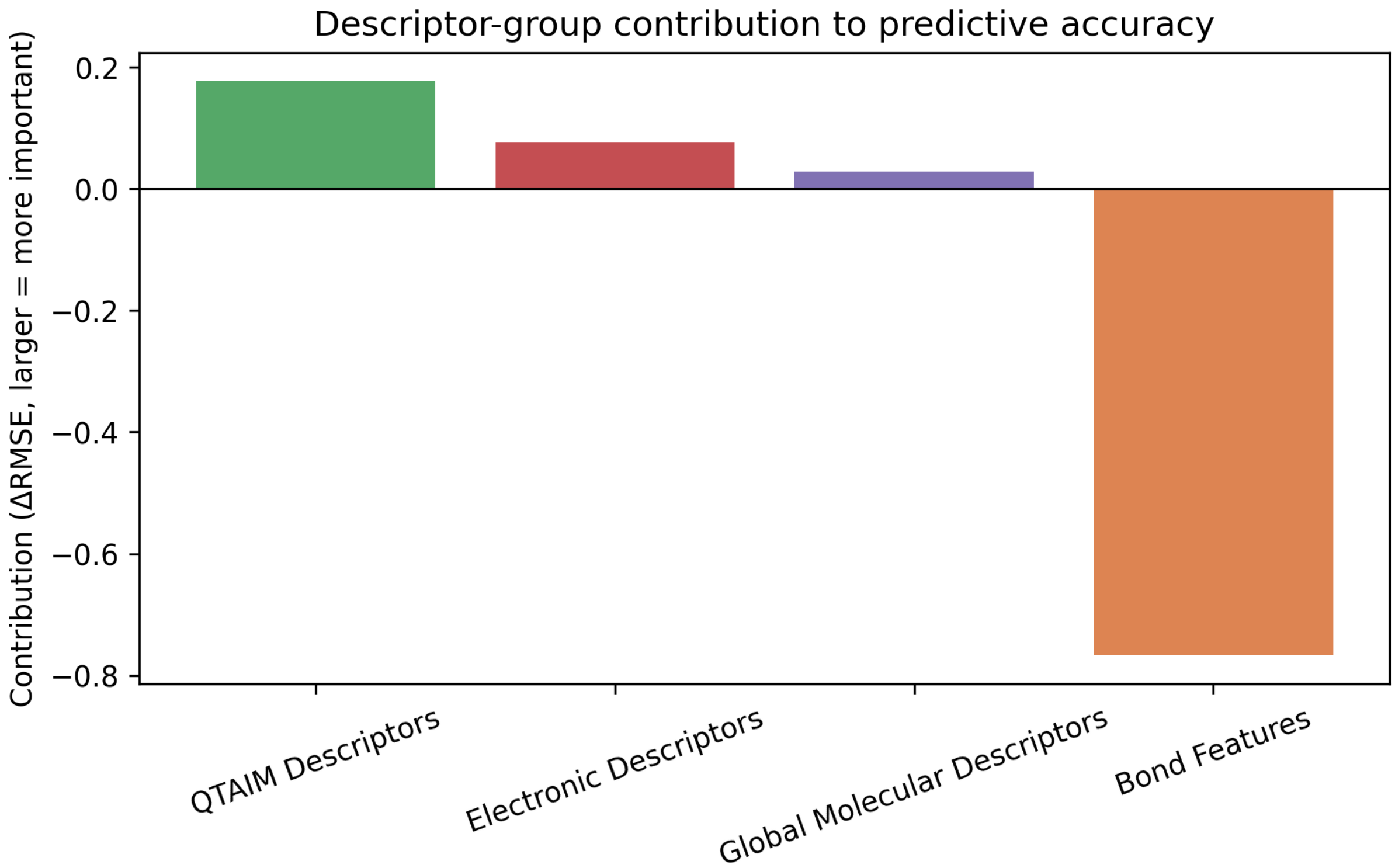

Figure SI IV: Contribution of the different descriptor groups to predictive accuracy. The contribution is expressed as the change in RMSE associated with each descriptor group; positive values indicate an increase in RMSE relative to the corresponding reference representation.

Taken together, the ablation and contribution analyses indicate that the QTAIM descriptors provide the most consistently beneficial complementary information to the graph topology. Electronic and global molecular descriptors provide smaller additional improvements, while the contribution of bond descriptors is strongly representation-dependent. The results therefore support the use of a compact topology + QTAIM representation when a reduced descriptor set is desired, while retaining the complete representation when maximizing interpolation accuracy is the primary objective.

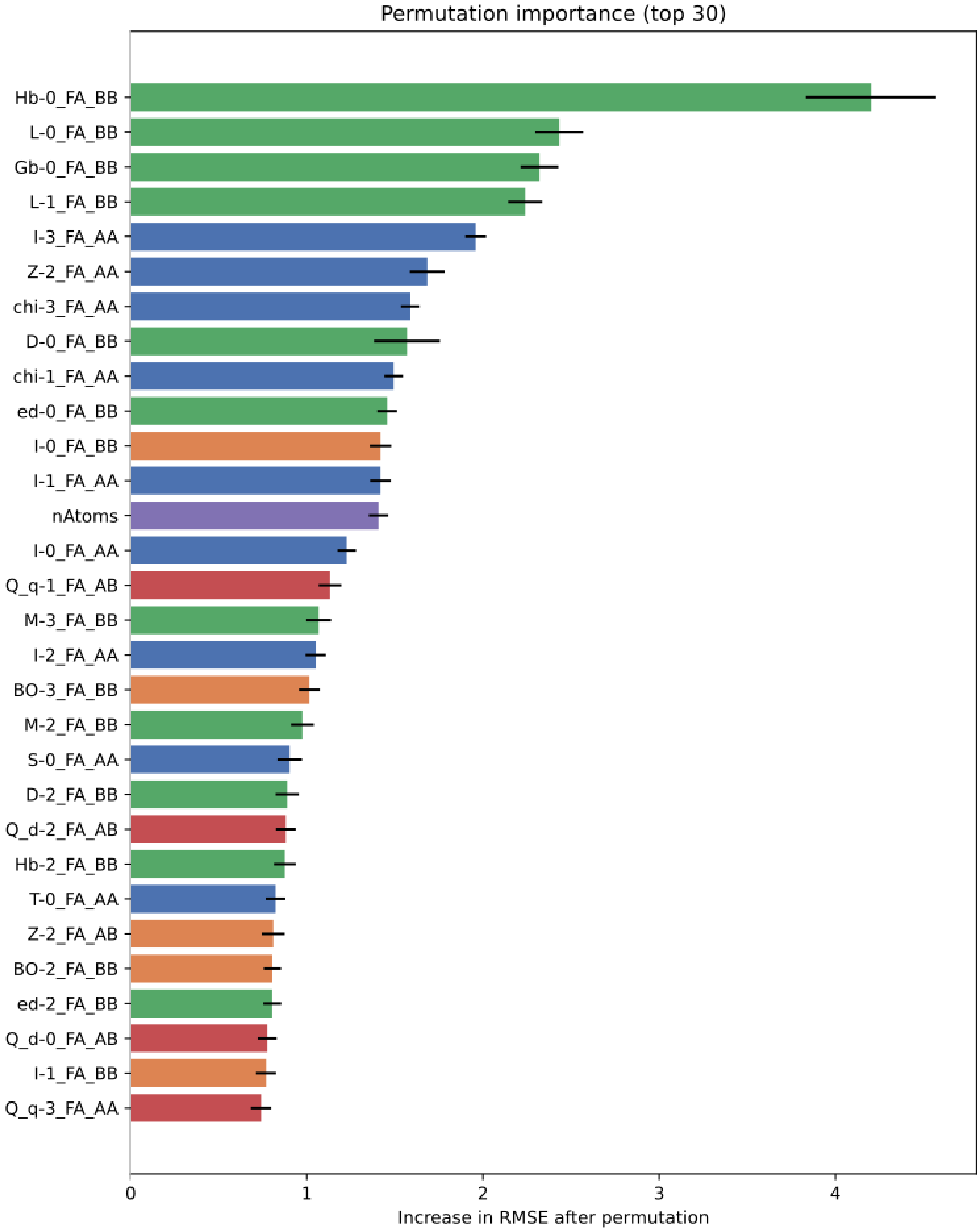

Figure SI V: Permutation feature importance for POMs-GibbsNet. The figure shows the 30 descriptors producing the largest increase in RMSE when individually permuted. Larger values indicate a greater dependence of the model predictions on the corresponding descriptor.

4 Generalization and validation

The predictive performance obtained from a random train–test split does not by itself establish generalization to chemically distinct polyoxometalate systems. Because the dataset contains multiple protonation states, isomers, and metal-substituted analogues belonging to the same parent chemical systems, a random split can place closely related structures in both the training and test sets. To assess transferability under more chemically demanding conditions, two complementary group-wise validation strategies were therefore employed: leave-one-system-out cross-validation (LOSO) and leave-one-metal-family-out cross-validation (LOMFO). The random split is retained as a measure of interpolation within the sampled chemical space, whereas the group-wise schemes provide a stricter assessment of generalization to chemically distinct systems.

The composition of each validation fold was explicitly recorded to ensure that the chemical groups withheld during testing were absent from the corresponding training set. The fold composition and associated system-level information are provided in the following sections.

4.1 Random split

As a reference measure of interpolation performance, the dataset was divided into training, validation, and test subsets using a random 70/15/15 split. The splitting procedure was performed at the molecular level, such that individual POM structures were assigned independently to the three subsets. The resulting test set therefore contains structures belonging to chemical systems that are also represented in the training set.

Under this protocol, POMs-GibbsNet achieves $R^2 = 0.993$ with an RMSE of approximately 1.9 $\mathrm{kcal\,mol^{-1}}$ on the held-out test set. The high performance obtained under this split demonstrates that the model can accurately interpolate within the chemical space represented in the training data. However, because chemically related structures can occur in both training and test subsets, these results should not be interpreted as evidence of transferability to previously unseen chemical systems.

The random-split results are therefore used as the baseline against which the more stringent chemically informed validation schemes are compared.

4.2 Leave-one-system-out cross-validation

To evaluate generalization to previously unseen chemical systems, leave-one-system-out cross-validation (LOSO) was performed across the 19 POM systems represented in the dataset. In each fold, all structures belonging to one parent chemical system were excluded from model development and used exclusively for testing. Consequently, all protonation states, structural variants, and metal-substituted analogues belonging to the withheld system were unavailable during training.

The LOSO results reveal a clear distinction between typical and worst-case transferability. Across the 19 held-out systems, the median test-set performance is $R^2 = 0.985$ and RMSE = 2.03 $\mathrm{kcal\,mol^{-1}}$. The corresponding mean values are strongly affected by a small number of difficult folds, giving $R^2 = -0.935 \pm 8.07$ and RMSE = 7.50 ± 19.29 $\mathrm{kcal\,mol^{-1}}$. The large difference between the mean and median therefore reflects a highly skewed fold distribution rather than uniformly poor generalization.

Most systems are predicted with RMSE values between approximately 0.9 and 3.0 $\mathrm{kcal\,mol^{-1}}$. Particularly good transfer is obtained for AsMoW (RMSE = 0.88 $\mathrm{kcal\,mol^{-1}}$), AsMo (1.17 $\mathrm{kcal\,mol^{-1}}$), AsW (1.18 $\mathrm{kcal\,mol^{-1}}$), and several of the mixed-metal systems. In contrast, the largest errors occur for the Mo, PMo, and W folds, with RMSE values of 16.98, 7.87, and 87.92 $\mathrm{kcal\,mol^{-1}}$,

respectively. The W fold is an extreme outlier, with $R^2 = -35.16$, whereas the Mo fold gives $R^2 = 0.949$ despite its very large RMSE. The corresponding fold-level results are summarized in Table SI VIII.

These failures demonstrate that high random-split performance does not guarantee uniform transferability to chemically distinct systems. In particular, the W and Mo systems are small single-metal isopolyanions and occupy regions of chemical and descriptor space that are poorly represented by the remaining systems. The LOSO results therefore support interpreting POMs-GibbsNet primarily as a reliable interpolator within the well-represented region of POM chemical space rather than as a general extrapolator.

Importantly, the LOSO analysis also shows that poor performance is not a universal consequence of withholding an entire chemical system. Seventeen of the nineteen folds retain $R^2 > 0.92$, and most exhibit RMSE values close to those obtained under the random split. The observed degradation is consequently chemically systematic and concentrated in a small number of systems rather than being an intrinsic limitation of system-level withholding.

Table SI VIII: Test-set performance for the 19 leave-one-system-out (LOSO) folds. In each fold, the complete POM system indicated in the first column was withheld from model development. RMSE and MAE are reported in kcal mol^{-1}.

**Withheld system**	**R^2**	**RMSE**	**MAE**
AsMo	0.995	1.17	0.93
AsMoW	0.995	0.88	0.61
AsTaW	0.981	1.65	1.19
AsW	0.995	1.18	0.87
Mo	0.949	16.98	11.78
Nb	0.995	2.04	1.46
PMo	0.753	7.87	2.60
PMoW	0.985	2.07	1.30
PTaW	0.990	1.65	1.23
PW	0.939	2.31	1.91
SiMo	0.989	1.65	1.16
SiMoW	0.976	2.24	1.68
SiTaW	0.981	1.93	1.58
SiW	0.989	1.59	1.34
Ta	0.990	3.01	2.31
V	0.928	2.81	2.39
VMo	0.984	2.03	1.74
VW	0.989	1.54	1.24
W	-35.162	87.92	19.51
**Median**	0.985	2.03	1.46
**Mean**	-0.935	7.50	2.99

4.3 Leave-one-metal-family-out cross-validation

A complementary leave-one-metal-family-out (LOMFO) validation was performed to assess transferability across metal identity. Whereas LOSO withholds one parent chemical system at a time, LOMFO removes all structures associated with a given metal family from model development and evaluates the model on structures containing that metal family. This provides

a more demanding test of whether the learned representation captures chemical relationships that can be transferred to metal identities not encountered during training.

Seven LOMFO folds were evaluated, corresponding to Mo, Nb, Ta, V, W, mixed-metal, and unknown metal families. Across these folds, the median test-set performance was $R^2 = 0.941$, RMSE = 4.28 kcal mol^{-1}, and MAE = 3.70 kcal mol^{-1}. The corresponding mean values were $R^2 = 0.837$, RMSE = 9.49 kcal mol^{-1}, and MAE = 5.05 kcal mol^{-1}. As in the LOSO analysis, the difference between the mean and median is caused by a small number of difficult folds rather than uniformly poor performance.

The Mo holdout represents the most severe failure, with $R^2 = 0.511$, RMSE = 38.73 kcal mol^{-1}, and MAE = 16.22 kcal mol^{-1}. The Nb and Ta holdouts are also substantially more difficult than the typical LOSO folds, with RMSE values of 5.87 and 9.16 kcal mol^{-1}, respectively. In contrast, withholding V, W, and the mixed-metal family gives RMSE values of 2.52, 3.43, and 2.44 kcal mol^{-1}, respectively. The unknown-metal fold gives an intermediate performance, with an RMSE of 4.28 kcal mol^{-1}.

The LOMFO results therefore provide a more stringent assessment of chemical transferability than the random split and, in several cases, than LOSO. In particular, the increase in error when metal identities are completely excluded from training demonstrates that part of the predictive performance depends on chemical information associated with the metal families represented in the training data. Nevertheless, the relatively good performance for several withheld families shows that the descriptor representation retains transferable information across different metal environments.

Taken together with the LOSO results, the LOMFO analysis indicates that POMs-GibbsNet should not be interpreted as a uniformly transferable model across the entire metal–oxo chemical space. Its strongest performance is obtained within regions containing chemically related training examples, while extrapolation to poorly represented metal families can lead to substantially larger errors. These results motivate the use of the applicability-domain and uncertainty analyses described in Section 5 when the model is applied to chemically novel systems.

Table SI IX: Test-set performance for the leave-one-metal-family-out (LOMFO) validation. Each fold corresponds to a metal family withheld from model development. RMSE and MAE are reported in kcal mol^{-1}.

**Withheld family**	$\mathbf{R^2}$	**RMSE**	**MAE**
Mo	0.511	38.73	16.22
Nb	0.962	5.87	4.71
Ta	0.903	9.16	4.38
V	0.941	2.52	2.08
W	0.954	3.43	2.39
Mixed-metal	0.973	2.44	1.84
Unknown	0.613	4.28	3.70
Median	0.941	4.28	3.70
Mean	0.837	9.49	5.05

5 Uncertainty quantification and applicability domain

Predictive accuracy alone does not establish whether an individual prediction is reliable, particularly when the model is applied to structures that differ substantially from those represented in the training data. To assess prediction reliability, two uncertainty-quantification strategies

were evaluated: Monte-Carlo dropout and deep ensembles. In parallel, a Mahalanobis-distance applicability-domain (AD) analysis was used to quantify the similarity of each structure to the multivariate descriptor distribution represented in the training data.

The uncertainty methods were compared using calibration error, negative log-likelihood, empirical coverage of nominal 95% prediction intervals, and the correlation between predicted uncertainty and observed absolute prediction error. The applicability-domain metric was evaluated separately as a measure of chemical-space similarity. The results are summarized in Table SI X.

Table SI X: Benchmark of uncertainty-quantification strategies for POMs-GibbsNet. ECE and MCE denote expected and maximum calibration error, respectively. Coverage_{95} is the empirical coverage of the nominal 95% prediction interval.

Method	ECE	MCE	NLL	Coverage_{95}	Pearson	Spearman
MC dropout	0.037	0.093	2.416	0.934	0.148	0.099
Deep ensemble	0.243	0.429	5.608	0.554	0.285	0.181

5.1 Monte-Carlo dropout uncertainty

Monte-Carlo dropout was selected as the primary uncertainty estimate for POMs-GibbsNet. During inference, dropout was retained in the trained network and multiple stochastic forward passes were performed for each structure. The mean of these predictions was used as the final estimate of $G - E$, while the standard deviation of the stochastic predictions was used as the uncertainty estimate.

The resulting uncertainty estimates are well calibrated for the present dataset. The empirical coverage of the nominal 95% prediction intervals is 93.4%, close to the target coverage, with an ECE of 0.037 and MCE of 0.093. These results indicate that the uncertainty scale is reasonably consistent with the observed distribution of prediction errors.

The uncertainty estimate should nevertheless be interpreted as a probabilistic indicator rather than as a direct prediction of the absolute error. The relatively modest Pearson and Spearman correlations between uncertainty and absolute error indicate that uncertainty does not uniquely determine the magnitude of an individual prediction error. Its principal value is therefore as a screening criterion for identifying predictions that warrant additional scrutiny.

The distribution of Monte-Carlo-dropout uncertainties across the test structures is shown in Figure SI VI. The distribution is concentrated around a median uncertainty of 0.410, with most structures exhibiting uncertainties between approximately 0.3 and 0.6. A smaller population of structures exhibits substantially larger uncertainties, extending beyond 1.0 and reaching approximately 1.6. These high-uncertainty predictions correspond to chemically less well represented regions of the dataset, as examined in the subsequent applicability-domain and chemical-space analyses.

5.2 Deep-ensemble uncertainty

Deep-ensemble uncertainty was evaluated as an alternative to Monte-Carlo dropout. Independent POMs-GibbsNet models were trained and the variation in their predictions was used to estimate predictive uncertainty.

For the present dataset, the deep ensemble is substantially less well calibrated than Monte-Carlo dropout. The ECE and MCE increase to 0.243 and 0.429, respectively, while the nominal 95% prediction intervals cover only 55.4% of the observations. The NLL is also considerably

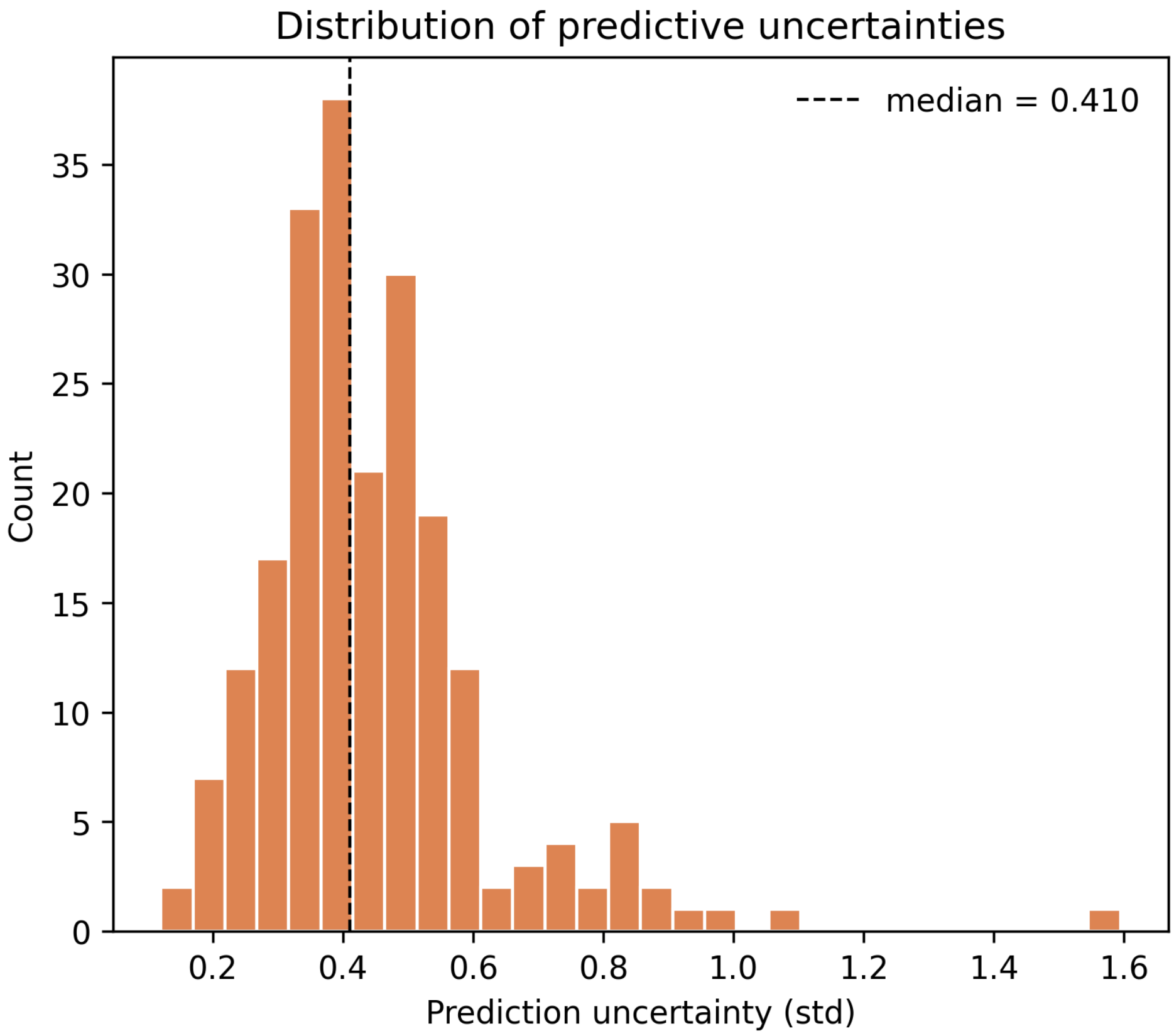

Figure SI VI: Distribution of Monte-Carlo-dropout predictive uncertainties across the test structures. The dashed line indicates the median uncertainty.

higher, at 5.61. These results indicate strong under-coverage of the nominal prediction intervals and therefore over-confidence in the raw ensemble uncertainty.

Although the deep ensemble shows somewhat stronger correlation between uncertainty and absolute error than Monte-Carlo dropout, this does not compensate for its poor calibration. Moreover, the ensemble requires approximately 48.3 s for the evaluated dataset, compared with 6.6 s for Monte-Carlo dropout. Deep ensembles were therefore not selected as the default uncertainty estimator for the workflow.

5.3 Applicability-domain analysis

An applicability-domain (AD) analysis was performed to determine whether the uncertainty estimates respond systematically when the model is required to predict structures outside the chemical space represented during training. The analysis was based on the Mahalanobis distance in descriptor space, which accounts for correlations between descriptors and provides a multivariate measure of the similarity between a test structure and the training distribution.

The Mahalanobis distance shows a moderate positive association with the observed absolute prediction error, with Pearson and Spearman correlations of 0.437 and 0.420, respectively. This indicates that descriptor-space distance contains useful information about prediction reliability, although it is not sufficiently strong to serve as a standalone predictor of the magnitude of an individual error.

A more direct test of the applicability-domain behaviour was obtained by withholding complete metal families from the training data and comparing the uncertainty and error of the resulting out-of-domain structures with those of in-domain structures. The results are summarized in Table SI XI. In every metal-family holdout, both the mean predictive uncertainty

and the mean absolute error increase relative to the corresponding in-domain population. The magnitude of this increase depends strongly on the withheld chemical family.

The largest uncertainty inflation is observed for the unknown family, for which the mean uncertainty increases by a factor of 9.14 and the MAE by a factor of 8.40. The W and Mo families show similarly pronounced effects, with uncertainty increases of 4.91 and 4.20, respectively, accompanied by MAE increases of 7.75 and 6.15. The Nb, Ta, and V families show more moderate but still systematic increases in uncertainty, by factors of 2.34, 2.22, and 2.10, respectively. Even the mixed-metal holdout, which represents the largest test population, shows an increase in uncertainty by a factor of 1.36 and in MAE by a factor of 1.39.

These results demonstrate that the uncertainty estimate responds systematically to chemical extrapolation. Structures outside the region represented by the training data tend to exhibit both larger predictive uncertainty and larger errors. The effect is particularly pronounced for the same metal families that show degraded performance in the LOSO and LOMFO analyses, providing an independent indication that the model's failures are associated with departures from the training distribution rather than with random fluctuations in prediction error.

The applicability-domain analysis therefore provides a practical mechanism for screening predictions before they are used downstream. Predictions associated with large descriptor-space distances and elevated Monte-Carlo-dropout uncertainty should be treated as potentially extrapolative and, where possible, subjected to additional DFT validation.

Table SI XI: Applicability-domain analysis for metal-family holdouts. OOD denotes structures belonging to the withheld metal family, while in-domain values correspond to structures represented by related metal families in the training distribution. Uncertainty and error ratios are calculated as OOD divided by in-domain values.

Family	$\sigma_{\mathrm{OOD}}/\sigma_{\mathrm{ID}}$	$\mathrm{MAE}_{\mathrm{OOD}}/\mathrm{MAE}_{\mathrm{ID}}$	N_{test}	N_{train}
Mo	4.20	6.15	163	1256
W	4.91	7.75	140	1279
Unknown	9.14	8.40	12	1407
Mixed-metal	1.36	1.39	983	436
Nb	2.34	1.68	40	1379
Ta	2.22	2.18	40	1379
V	2.10	2.83	41	1378

5.4 Uncertainty across chemical space

The distribution of Monte-Carlo-dropout uncertainty was further examined as a function of metal composition, parent POM system, cluster size, and molecular charge. These analyses provide a chemical interpretation of the uncertainty estimates and help identify regions in which the model is less well constrained by the available training data.

Metal composition has a clear effect on the predicted uncertainty. The rare metal families Ta and Nb show mean uncertainties of 0.716 and 0.543, respectively, compared with 0.405 for mixed-metal systems. V also exhibits elevated uncertainty (0.400), although its small sample size limits the strength of this comparison. The corresponding mixed-metal systems, which represent approximately 68% of the evaluated structures, generally exhibit lower uncertainty. This trend is consistent with the validation results showing that model performance is strongest in the densely sampled mixed-metal region of chemical space.

At the individual-system level, Ta shows the highest mean uncertainty (0.716), followed by W (0.627), Mo (0.608), and SiTaW (0.547). In contrast, the densely represented AsMoW

and AsTaW systems show mean uncertainties of only 0.387 and 0.387, respectively. These differences indicate that uncertainty is related to chemical representation and system coverage rather than being uniformly distributed across the dataset.

Cluster size shows a comparatively weak dependence. Mean uncertainties range from 0.393 to 0.498 across the four cluster-size bins, with no monotonic increase with molecular size. This observation is important because it indicates that larger POMs are not intrinsically associated with greater predictive uncertainty. The main limitations of the model therefore arise from chemical composition and descriptor-space coverage rather than cluster size itself.

The charge-stratified analysis reveals substantially larger uncertainties at several extreme charge states. The highest values occur for $q = -14$ (uncertainty = 1.59), $q = +2$ (0.99), and $q = -16$ (0.84). However, these charge states contain only one structure each and should therefore be regarded as individual outliers rather than statistically established charge-dependent trends. The more populated charge states between -1 and -8 exhibit considerably more uniform uncertainties, generally in the range of approximately 0.39–0.50.

Overall, the uncertainty analysis identifies chemical composition and representation-space coverage as more important determinants of predictive reliability than molecular size. This behaviour is consistent with the LOSO and LOMFO validation results and supports the use of uncertainty estimates and applicability-domain distances as complementary indicators when applying POMs-GibbsNet to chemically novel structures.

The dependence of predictive uncertainty on POM family is shown in Figure SI VII. The distributions reveal substantially different uncertainty levels between chemical families. Ta exhibits the highest typical uncertainty, followed by Nb, Mo, and W, whereas the mixed-metal systems show a lower and narrower uncertainty distribution. The same qualitative ordering is observed in the mean uncertainties reported in Table SI XII.

The system-resolved uncertainty distributions are summarized in Table SI XII. The highest mean uncertainties are observed for Ta (0.716), W (0.627), and Mo (0.608), whereas the densely represented mixed-metal systems AsMoW and AsTaW show substantially lower mean uncertainties of 0.387 and 0.387, respectively. The system-level results therefore support the conclusion that predictive uncertainty is primarily associated with chemical representation and system coverage rather than with cluster size alone.

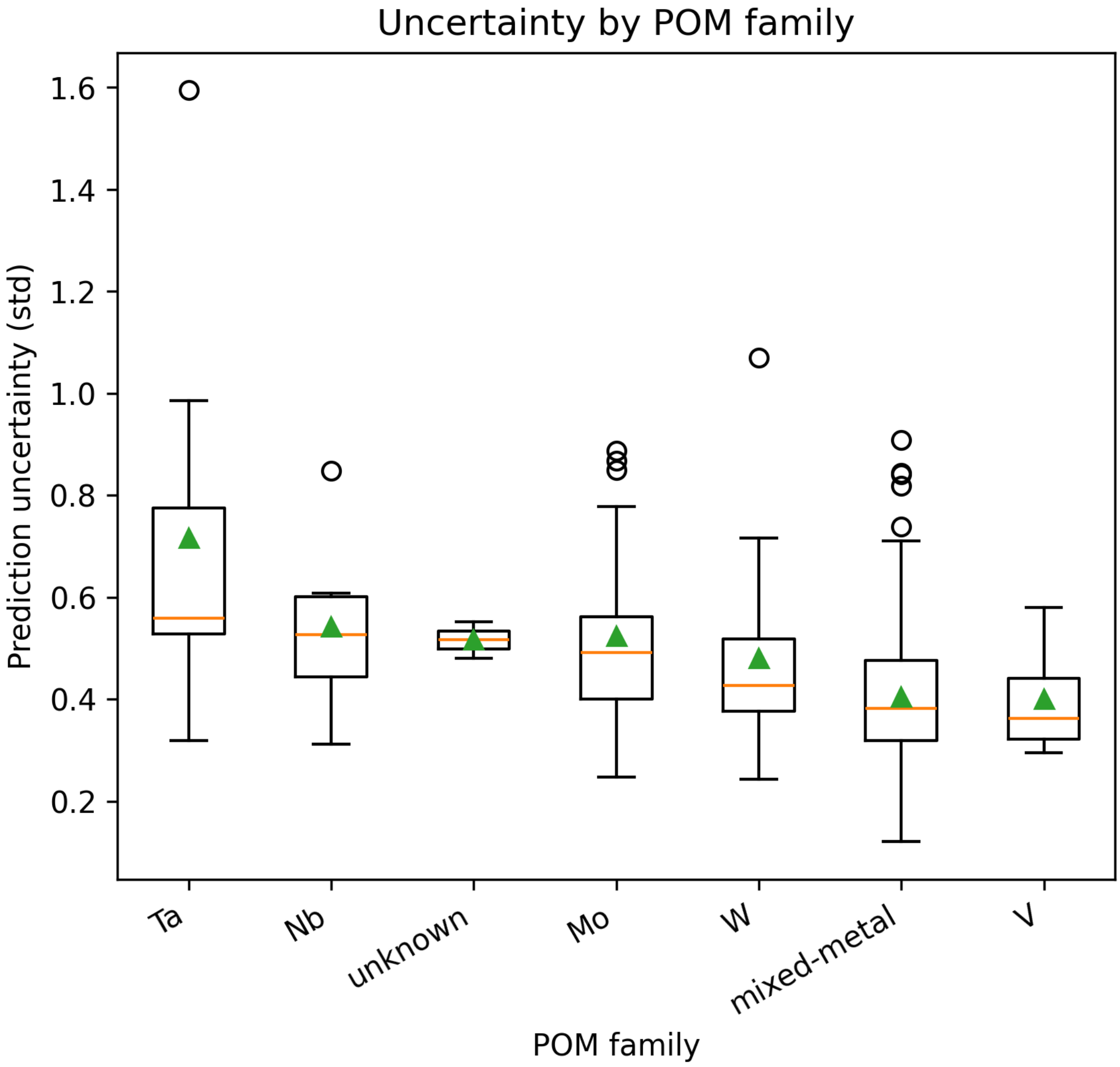

Figure SI VII: Distribution of Monte-Carlo-dropout predictive uncertainty across POM families. Triangles indicate the mean uncertainty and circles denote individual high-uncertainty observations.

Table SI XII: Predictive uncertainty and absolute error by POM system. N is the number of test structures, σ_{unc} is the standard deviation of the Monte-Carlo-dropout predictive distribution, and MAE is the absolute prediction error in $\mathrm{kcal\,mol^{-1}}$. The fraction gives the proportion of the complete dataset represented by each system.

**System**	$\overline{\boldsymbol{\sigma}}$	**median($\boldsymbol{\sigma}$)**	$\boldsymbol{\sigma(\sigma)}$	$\overline{\mathbf{\lvert error\rvert}}$	**median($\lvert$error$\rvert$)**
AsMo	0.448	0.485	0.062	0.800	0.394
AsMoW	0.387	0.370	0.129	0.635	0.489
AsTaW	0.387	0.388	0.138	0.715	0.527
AsW	0.448	0.425	0.149	0.795	0.626
Mo	0.608	0.541	0.203	1.656	1.385
Nb	0.543	0.526	0.184	0.555	0.599
PMo	0.458	0.493	0.119	0.934	0.771
PMoW	0.422	0.380	0.155	0.992	0.816
PTaW	0.428	0.434	0.101	1.145	0.700
PW	0.442	0.442	0.056	1.469	1.469
SiMo	0.508	0.481	0.133	2.295	2.115
SiMoW	0.436	0.389	0.152	1.469	1.536
SiTaW	0.547	0.476	0.244	1.523	1.637
SiW	0.420	0.420	0.077	1.086	1.086
Ta	0.716	0.559	0.380	1.218	1.068
V	0.435	0.395	0.129	1.410	1.195
VMo	0.419	0.403	0.107	1.864	1.991
VW	0.389	0.379	0.075	2.405	2.464
W	0.627	0.518	0.302	1.451	0.777

6 Error analysis and limitations

The validation analyses demonstrate that POMs-GibbsNet achieves strong performance within the well-represented region of the dataset, while the uncertainty and applicability-domain analyses in Section 5 identify regions in which predictions become less reliable. To determine the chemical origin of these deviations, a detailed error analysis was performed by grouping predictions according to metal composition, parent POM system, cluster size, molecular charge, and other structural characteristics.

The purpose of this analysis is not to redefine the overall model performance, but to identify systematic failure modes and distinguish them from isolated large errors. Particular attention was therefore given to the systems and structures associated with the largest prediction errors and to their relation to the chemically informed validation results.

6.1 Error by chemical system

The distribution of prediction errors across chemical systems is highly non-uniform. Mixed-metal systems are predicted substantially more accurately than single-metal systems, with reaction-level RMSE values of 1.41 and 4.17 $\mathrm{kcal\,mol^{-1}}$, respectively. The best-performing systems are the densely represented mixed-metal families AsMoW and AsTaW, with RMSE values of 0.84 and 1.02 $\mathrm{kcal\,mol^{-1}}$, respectively.

The largest errors are concentrated in a small number of single-metal systems. Mo is the most difficult system, with RMSE = 11.41 $\mathrm{kcal\,mol^{-1}}$, MAE = 6.34 $\mathrm{kcal\,mol^{-1}}$, and $R^2 = 0.9496$, followed by W (RMSE = 3.65 $\mathrm{kcal\,mol^{-1}}$), Nb (2.90 $\mathrm{kcal\,mol^{-1}}$), and Ta (2.84 $\mathrm{kcal\,mol^{-1}}$). This ordering is consistent with the LOSO and LOMFO analyses, in which Mo and W are among the most difficult chemical families to predict when withheld from model development.

The difference between single- and mixed-metal systems should not, however, be interpreted simply as an effect of metal count. The mixed-metal systems constitute the most densely sampled region of the dataset and contain numerous chemically related structures. Their improved performance therefore reflects, at least in part, the greater representation of similar chemical environments in the training data. Conversely, the problematic single-metal systems occupy less densely populated regions of descriptor space and exhibit higher predictive uncertainty.

Importantly, the relationship between data abundance and prediction error is not sufficient to explain the observed differences. The correlation between training-set size and per-system error is negative but not statistically significant (Spearman $\rho = -0.205$, $p = 0.40$ for the extended representation). The failures therefore cannot be attributed to sample size alone and instead reflect a combination of chemical character and representation-space coverage.

6.2 Failure modes and difficult structures

A targeted analysis of the largest prediction errors identified a small number of particularly difficult structures. Thirty reactions were classified as difficult according to the predefined error criterion, and all thirty involve Mo condensation reactions. The largest reaction error is 41.3 $\mathrm{kcal\,mol^{-1}}$. Importantly, these apparently independent reaction failures can be traced to a single molecular structure, Mo05O22-7H. The thermal free-energy correction of this molecule is mispredicted by approximately 35.0 $\mathrm{kcal\,mol^{-1}}$, and the same molecular error propagates into multiple reactions in which the structure participates. This demonstrates that large reaction-level errors do not necessarily originate from multiple independent failures of the model; a single poorly predicted molecular correction can propagate through the reaction network.

The problematic structure is a highly protonated Mo species with a water-rich composition. Its occurrence in multiple reactions therefore amplifies the practical impact of an individual

molecular prediction error. The result provides a chemically interpretable example of how the limitations identified by LOSO and LOMFO can propagate into downstream calculations. This analysis also illustrates the value of the applicability-domain and uncertainty estimates. Structures associated with unusual chemical compositions or poorly represented regions of descriptor space can be flagged before their predictions are propagated through a reaction network, providing an opportunity for targeted DFT validation.

6.3 Chemical characteristics associated with prediction error

Several additional group-wise analyses were performed to determine whether prediction errors could be associated with simple molecular characteristics. Cluster size shows little evidence of being a dominant failure mode. Large clusters containing 44–106 atoms have a predictive uncertainty of approximately 0.44, compared with 0.45 for small clusters containing 6–25 atoms. At the reaction level, the corresponding RMSE values are 2.36 and 2.47 kcal mol^{-1}, respectively. Thus, the model does not systematically become less reliable as the number of atoms increases within the range represented by the dataset.

Molecular charge provides a more heterogeneous picture. The largest predictive uncertainties occur at several extreme charge states, including $q = -14$ ($\sigma = 1.59$), $q = +2$ ($\sigma = 0.99$), and $q = -16$ ($\sigma = 0.84$). However, these charge states contain very few structures, in some cases only one, and should therefore be regarded as indicators of potentially unusual chemical environments rather than as statistically established charge trends. The more highly populated charge states between -1 and -8 exhibit a much more uniform uncertainty distribution. The combined analysis therefore indicates that chemical composition, metal identity, protonation, and representation-space coverage are more informative indicators of prediction difficulty than cluster size alone.

6.4 Limitations and applicability

The error analysis defines the practical scope of POMs-GibbsNet. The model provides highly accurate predictions within the densely represented region of the training distribution, but its performance can deteriorate substantially when applied to chemically distinct systems or metal families. In particular, the Mo and W systems demonstrate that high performance under a random split cannot be interpreted as evidence of uniform extrapolative capability. The model should therefore be regarded primarily as an interpolation model within the represented POM chemical space. Application to structures containing poorly represented metal families, unusual protonation states, or other descriptor combinations far from the training distribution should be accompanied by the Monte-Carlo-dropout uncertainty and applicability-domain assessment described in Section 5.

The analysis also identifies several priorities for future dataset expansion. Additional data for the small single-metal isopolyanions, particularly Mo and W systems, would directly address the most severe observed failures. More examples of unusual charge states and hydration reactions would improve the statistical basis for assessing these regions of chemical space. Finally, targeted calculations of structures identified simultaneously as high-uncertainty and out-of-domain would provide an efficient strategy for expanding the training set in regions where the current model is least reliable.

These limitations do not undermine the use of POMs-GibbsNet as a surrogate for the thermal free-energy correction within its demonstrated domain. Rather, they define the conditions under which the predicted corrections should be considered reliable and provide a quantitative basis for identifying cases requiring additional electronic-structure calculations.

7 POMSimulator validation

7.1 Reaction-network construction

The practical utility of POMs-GibbsNet was evaluated by replacing the DFT thermal free-energy corrections within the POMSimulator workflow. For every optimized structure, the electronic energy obtained from DFT was combined with either the DFT-computed thermal correction or the value predicted by POMs-GibbsNet,

$$G = E_{\text{elec}} + G_{\text{corr}},$$

where G_{corr} denotes either the reference DFT correction or the machine-learned prediction. These corrected Gibbs free energies were then propagated through the complete reaction network generated by POMSimulator.

POMSimulator automatically constructs chemically balanced reaction networks by combining all molecular species within a given chemical system and identifying valid transformations through graph isomorphism, atom balance, and stoichiometric analysis. The reaction Gibbs free energy is computed as

$$\Delta G_{\text{rxn}} = \sum_i \nu_i G_i,$$

where ν_i is the stoichiometric coefficient of species i. The resulting reaction energies constitute the thermodynamic input used to compute equilibrium constants, construct reaction networks, and generate speciation diagrams.

Across the nineteen chemical systems investigated in this work, the complete network comprises 1 618 molecular species connected by 10 819 reactions, including 6 944 acid–base equilibria, 2 201 condensation reactions, 1 668 addition reactions, and 6 hydration reactions.

Table SI XIII: Composition of the reaction network generated by POMSimulator for the complete dataset. The network comprises 1 618 molecular species connected through 10 819 chemically balanced reactions classified according to their transformation type.

**Reaction type**	**Number of reactions**	**Fraction (%)**
Acid–base	6 915	63.9
Condensation	2 261	20.9
Addition	1 484	13.7
Hydration	159	1.5
**Total**	**10 819**	**100.0**

7.2 Reaction-level validation methodology

Unlike most machine-learning energy models, which are evaluated using absolute molecular energies, the quantity of interest for POMSimulator is the reaction free energy. Because reaction energies are obtained as linear combinations of several molecular Gibbs energies, molecular prediction errors may either accumulate or cancel depending on their sign and magnitude.

To quantify this behavior, molecular prediction errors were propagated through the complete reaction network. For each reaction, the accumulated molecular error,

$$E_{\text{acc}} = \sum_r |e_r|,$$

was compared with the resulting reaction error,

$$E_{\text{rxn}} = \left| \sum_r e_r \right| ,$$

where e_r is the signed prediction error of species r, weighted by its stoichiometric coefficient. The degree of cancellation was quantified through the cancellation factor,

$$CF = \frac{|\sum_r e_r|}{\sum_r |e_r|},$$

where $CF = 0$ corresponds to complete error cancellation and $CF = 1$ indicates no cancellation. This metric provides a direct measure of how effectively molecular prediction errors compensate when propagated to reaction thermodynamics.

The resulting distribution of reaction-energy prediction errors over the complete reaction network is shown in Figure SI VIII. Although individual molecular predictions exhibit finite errors, the reaction-energy errors remain tightly centered around zero, indicating that the model introduces little systematic bias once propagated through chemical reactions.

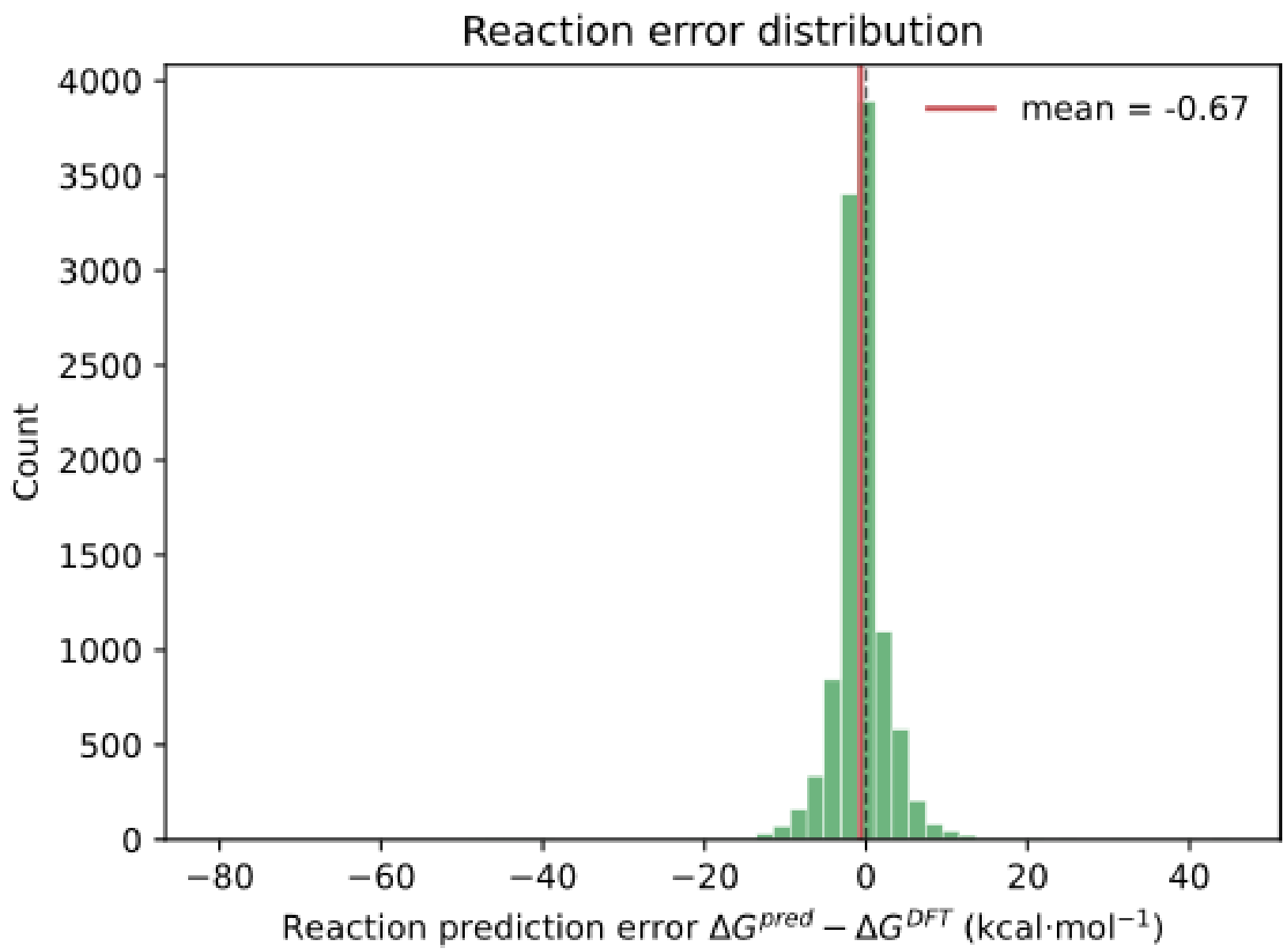

Figure SI VIII: Distribution of signed reaction free-energy prediction errors, $\Delta G^{\text{pred}} - \Delta G^{\text{DFT}}$, computed over the complete reaction network. The vertical red line indicates the mean signed error, while the dashed green line denotes zero prediction error.

To investigate whether prediction accuracy depends on reaction chemistry, reaction errors were further grouped according to reaction class (acid–base, addition, condensation and hydration). Summary statistics are reported in Table SI XVI, while the corresponding distributions are shown in Figure SI IX.

7.3 Error cancellation analysis

The reaction-level validation confirms that molecular prediction errors do not propagate linearly through the reaction network. Instead, positive and negative errors frequently compensate one another, resulting in substantially smaller reaction-energy errors than would be expected from the individual molecular predictions.

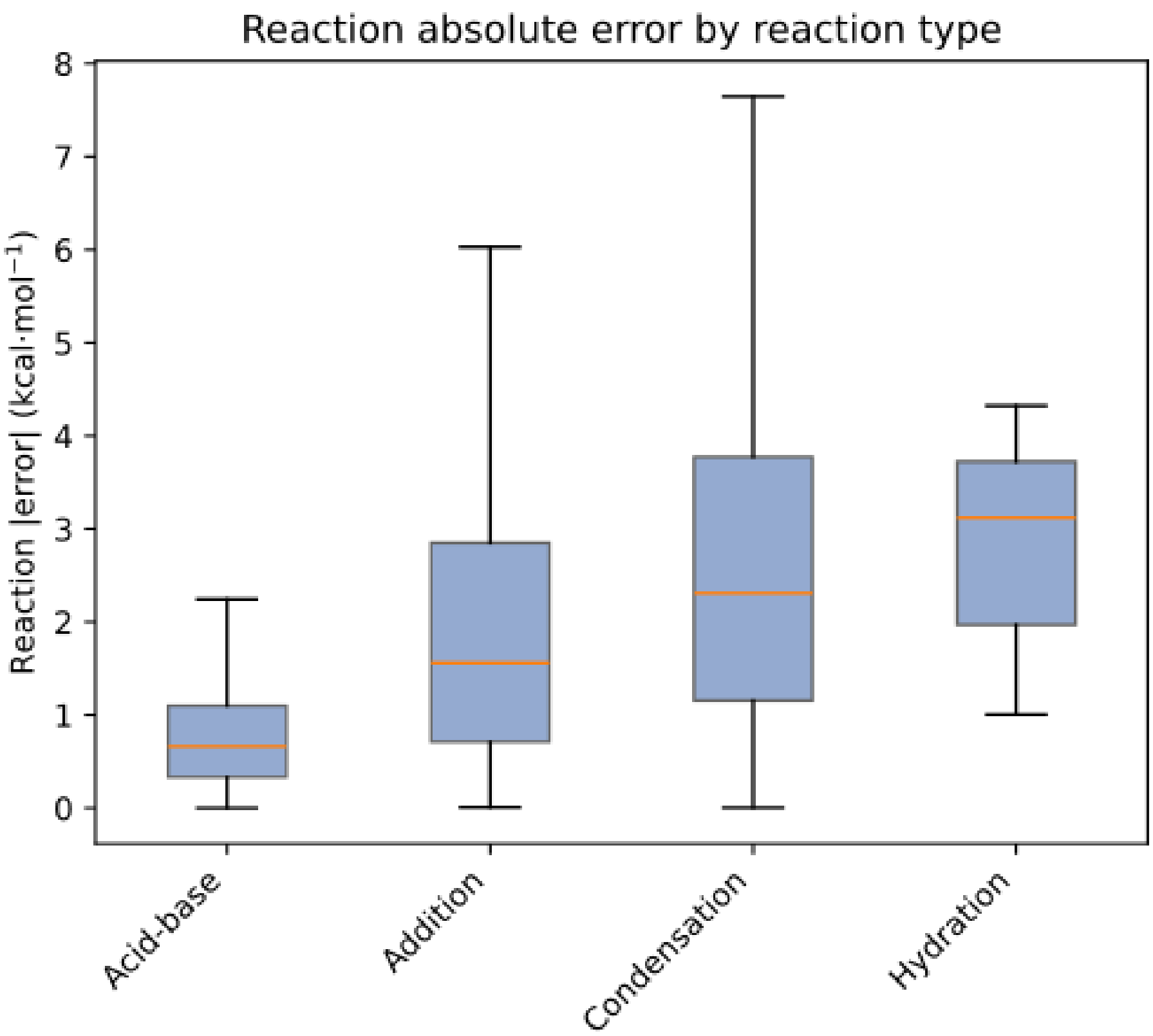

Figure SI IX: Distribution of absolute reaction free-energy prediction errors for the four reaction classes identified by POMSimulator: acid–base, addition, condensation, and hydration. Boxes represent the interquartile range, the horizontal line indicates the median, and whiskers extend to 1.5 times the interquartile range.

Figure SI XII summarizes the distribution of the cancellation factor (CF) over the complete network of 10 819 reactions. The distribution is strongly skewed toward low values, indicating that partial error cancellation is the dominant behaviour rather than the exception. The mean cancellation factor is 0.597 (median 0.605), corresponding to an average reduction of approximately 40% relative to the accumulated molecular error. Under the assumption of statistically independent molecular errors, the expected accumulated error would be 2.99 kcal mol^{-1}, whereas the observed mean reaction error is only 1.20 kcal mol^{-1}, demonstrating that the network benefits from substantial systematic error compensation.

The statistical significance of this reduction was evaluated by comparing, for every reaction, the accumulated molecular error with the corresponding reaction error. Both a paired one-sided Student's t-test and a Wilcoxon signed-rank test reject the null hypothesis of equal errors ($t = -64.9$, $p < 10^{-300}$), with a moderate paired effect size ($d_z = -0.62$) and a large rank-biserial correlation (-0.85). These results demonstrate that the observed reduction in reaction error is highly unlikely to arise from random fluctuations.

The extent of cancellation is nevertheless heterogeneous across the reaction network. Approximately 42.8% of reactions exhibit $CF < 0.5$, indicating that more than half of the accumulated molecular error is cancelled, while 24.5% satisfy $CF < 0.25$ and 10.3% achieve almost complete cancellation ($CF < 0.1$). Conversely, a smaller fraction of reactions shows little or no compensation ($CF \approx 1$), reflecting cases where molecular errors reinforce rather than cancel.

Although reaction and molecular errors remain correlated (Pearson correlation coefficient 0.84), the considerably lower Spearman correlation (0.53) indicates that error cancellation substantially alters the ranking of the most difficult predictions. Consequently, molecular pre-

Table SI XIV: Prediction statistics for reaction Gibbs free energies computed over the complete POMSimulator reaction network. Molecular prediction errors are propagated through the reaction stoichiometry and compared with DFT reference reaction energies.

**Metric**	**Value**	**Units**
Number of reactions	10 819	–
Mean signed error (MSE)	−0.67	$\mathrm{kcal\,mol^{-1}}$
Median absolute error	1.03	$\mathrm{kcal\,mol^{-1}}$
Mean absolute error	1.48	$\mathrm{kcal\,mol^{-1}}$
95th percentile absolute error	5.21	$\mathrm{kcal\,mol^{-1}}$
Maximum absolute error	25.0	$\mathrm{kcal\,mol^{-1}}$
Pearson correlation[a]	0.656	–
Spearman correlation[a]	0.573	–

[a]Correlation between accumulated molecular error and reaction error.

diction accuracy alone is not sufficient to identify the reactions with the largest thermodynamic uncertainty, highlighting the importance of evaluating surrogate models directly at the reaction level.

Figure SI XIII further stratifies the cancellation factor according to reaction class. The corresponding reaction-specific statistics are reported in Table SXX and discussed in the following subsection.

Table SI XV: Summary statistics of the reaction error-cancellation analysis. The cancellation factor is defined as $|\Delta G_{\mathrm{err}}|/\sum_i |e_i|$, where values below one indicate partial cancellation of molecular prediction errors.

**Statistic**	**Value**	**Interpretation**
Median cancellation factor	0.63	Typical reaction retains 63% of accumulated molecular error
Mean cancellation factor	0.61	Average cancellation across all reactions
Reactions with CF < 0.5	38.4%	Strong error cancellation
Reactions with CF < 1	96.8%	Some degree of cancellation
Pearson correlation	0.656	Molecular vs reaction error
Spearman correlation	0.573	Rank correlation

7.4 Reaction-class performance

The predictive accuracy of the machine-learned thermodynamic corrections depends on the type of chemical transformation considered. Table SXX summarizes the performance metrics for each reaction class, including the coefficient of determination (R^2), root-mean-square error (RMSE), mean absolute error (MAE), and the mean cancellation factor. Figure SI XIII complements these statistics by showing the full distribution of cancellation factors for each reaction class.

Acid–base reactions represent by far the largest subset of the reaction network (6 944 of the 10 819 reactions) and constitute the most accurately predicted class. Their reaction energies are reproduced with an RMSE of 1.14 $\mathrm{kcal\,mol^{-1}}$, an MAE of 0.73 $\mathrm{kcal\,mol^{-1}}$, and $R^2 = 0.99993$. This level of accuracy is particularly important because protonation equilibria dominate the speciation diagrams generated by POMSimulator and therefore determine the calculated solution composition over a broad pH range.

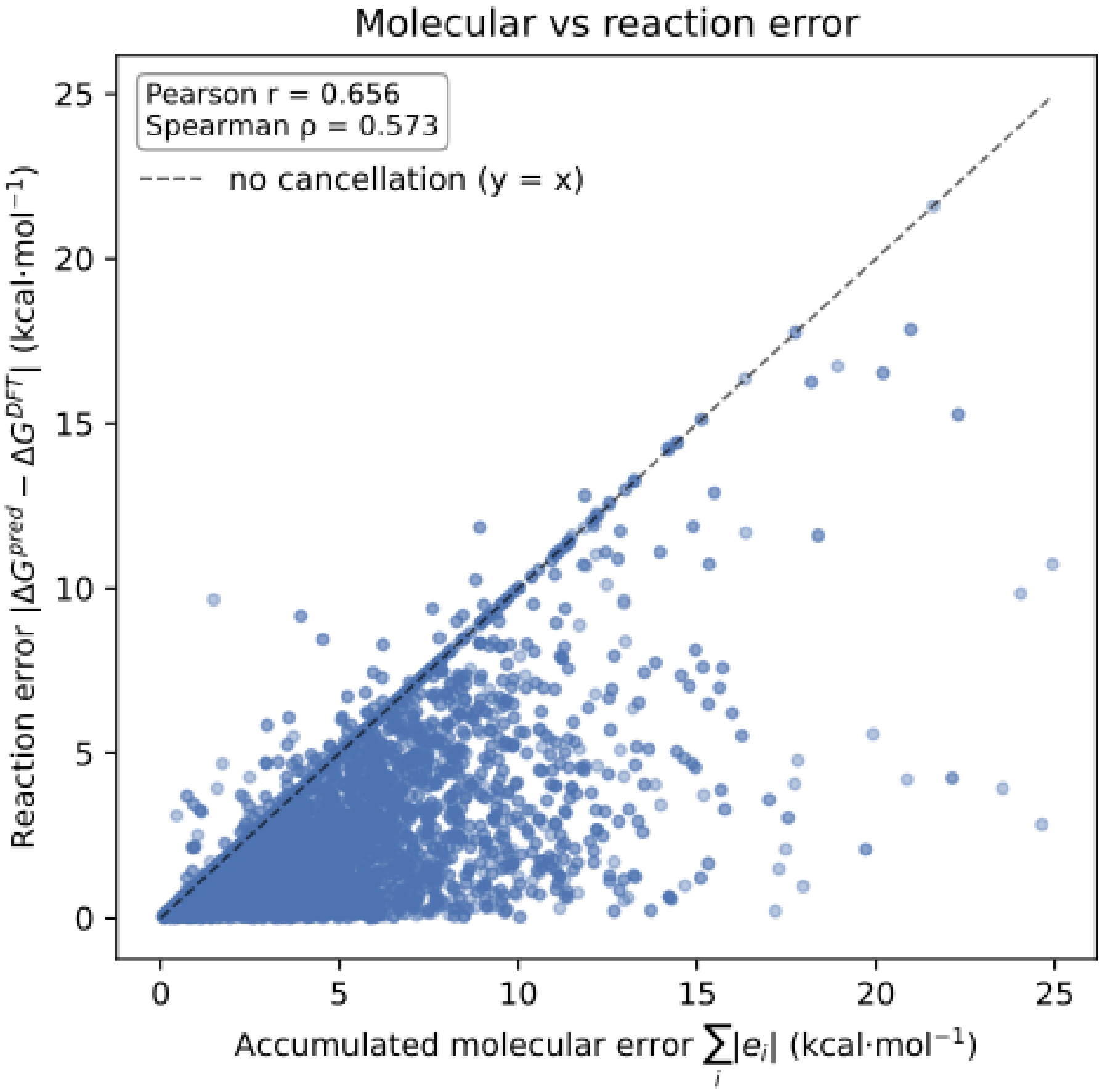

Figure SI X: Relationship between the accumulated molecular prediction error, $\sum_i |e_i|$, and the corresponding reaction free-energy prediction error, $|\Delta G^{\text{pred}} - \Delta G^{\text{DFT}}|$, for all reactions generated by POMSimulator. The dashed line represents the limiting case in which molecular errors accumulate without cancellation ($y = x$). Pearson and Spearman correlation coefficients between both quantities are reported in the inset.

Reactions involving structural rearrangements of the metal–oxo framework are consistently more challenging. Addition reactions ($n = 1\,668$) exhibit an RMSE of 2.66 kcal mol^{-1} and an MAE of 1.96 kcal mol^{-1} ($R^2 = 0.9964$), whereas condensation reactions ($n = 2\,201$) constitute the most difficult class, with an RMSE of 4.27 kcal mol^{-1} and an MAE of 2.13 kcal mol^{-1} ($R^2 = 0.9863$). These larger errors are consistent with the greater structural and electronic changes associated with the formation or cleavage of metal–oxygen bonds.

Only six hydration reactions are present in the current dataset. Although their absolute prediction errors remain modest (RMSE = 1.65 kcal mol^{-1}), they display the weakest error cancellation of all reaction classes, with a mean cancellation factor of 0.769 and a median value of 1.00. Owing to the very limited sample size, these observations should be regarded as preliminary rather than statistically representative.

Interestingly, the ranking of reaction classes according to cancellation factor differs from the ranking based on predictive accuracy. Condensation reactions exhibit the strongest average cancellation (mean $CF = 0.578$), despite having the largest reaction-energy errors. This behaviour reflects the fact that these reactions also accumulate the largest molecular prediction errors before cancellation occurs. Consequently, a low cancellation factor does not necessarily imply high predictive accuracy; rather, it quantifies the fraction of accumulated molecular error that is eliminated during propagation to the reaction level.

Beyond absolute prediction errors, the preservation of chemically relevant trends is of pri-

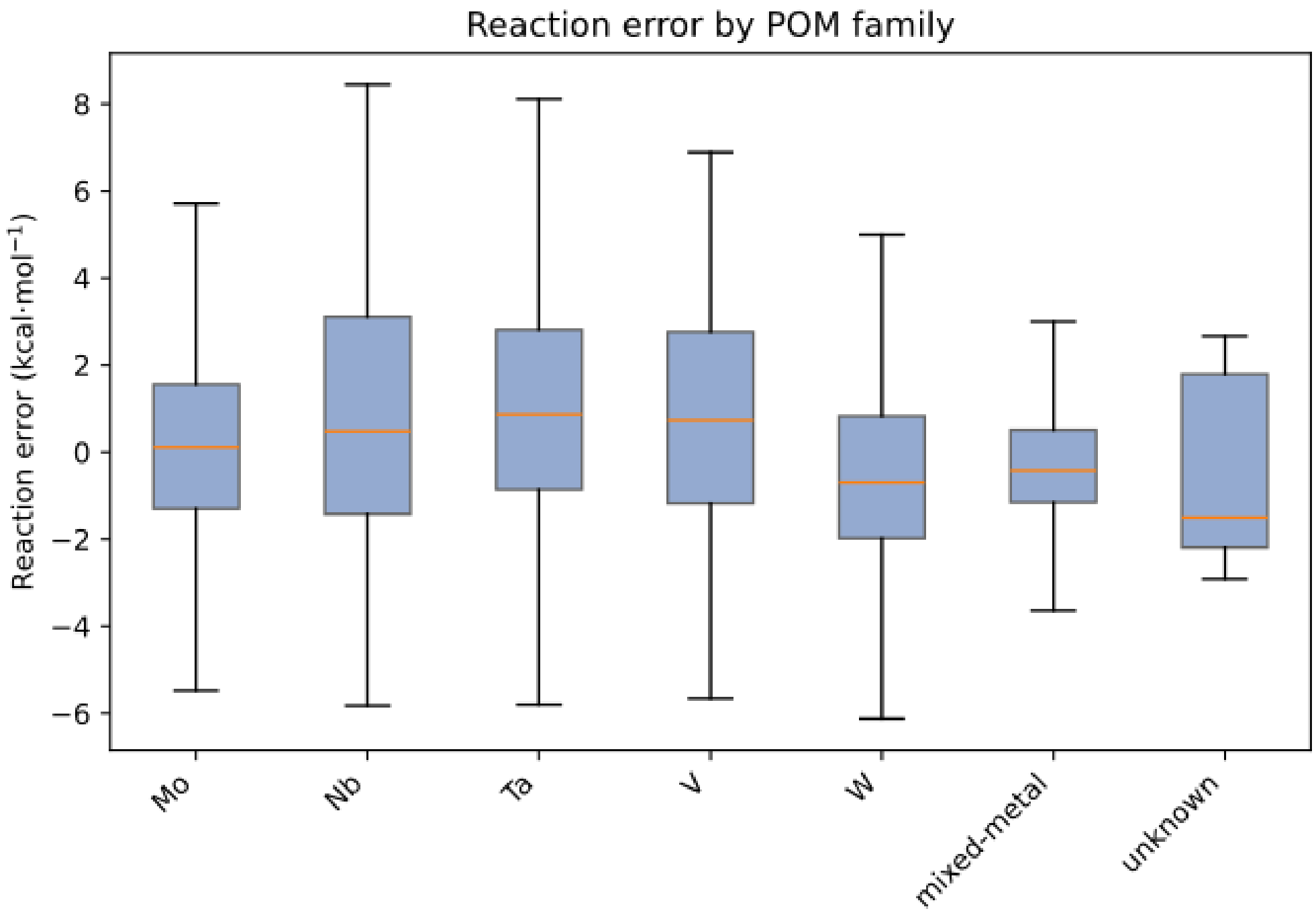

Figure SI XI: Distribution of signed reaction free-energy prediction errors grouped according to the POM family associated with each reaction. Boxes represent the interquartile range, the horizontal line indicates the median, and whiskers extend to 1.5 times the interquartile range.

mary importance for speciation calculations. Across the complete reaction network, the sign of the reaction Gibbs free energy is correctly reproduced for 97.8% of reactions, with individual systems ranging from 92.5% (Mo) to complete agreement for several mixed-metal systems. Likewise, the ordering of reaction energies within each chemical system is preserved with a mean Spearman correlation coefficient of 0.992, indicating that the relative thermodynamic landscape explored by POMSimulator remains essentially unchanged after replacing DFT vibrational thermochemistry with the machine-learned corrections.

Table SI XVI: Reaction prediction errors grouped by reaction class. Median absolute reaction errors and cancellation factors were computed for every reaction type in the complete POMSimulator reaction network.

**Reaction type**	**Median $\lvert\Delta G_{\mathrm{err}}\rvert$ (kcal mol^{-1})**	**Median cancellation factor**
Acid–base	0.60	0.61
Addition	1.56	0.61
Condensation	2.31	0.58
Hydration	3.09	1.00

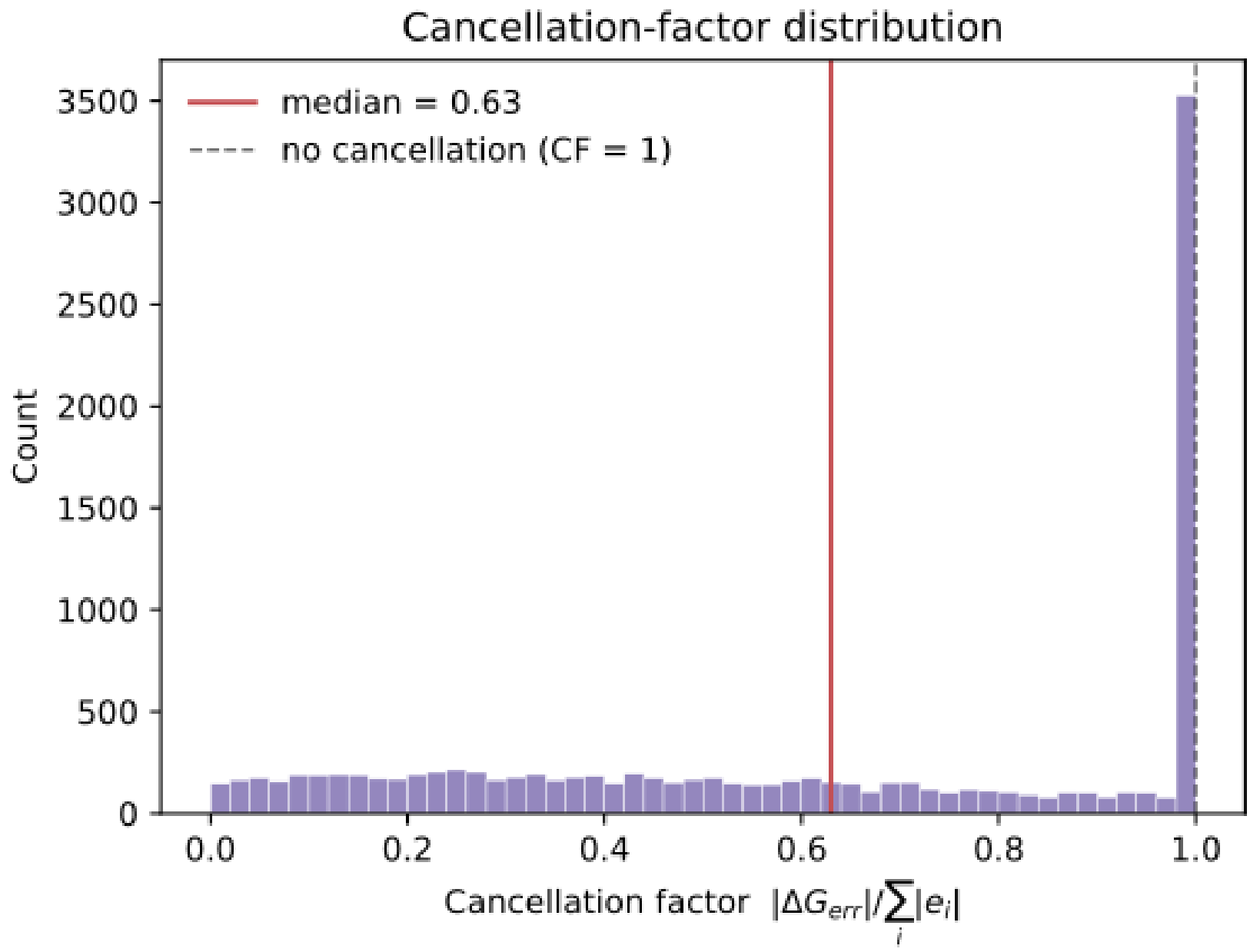

Figure SI XII: Distribution of the cancellation factor,computed for all reactions in the network. The solid red line marks the median cancellation factor, whereas the dashed gray line corresponds to $CF = 1$, indicating the absence of error cancellation.

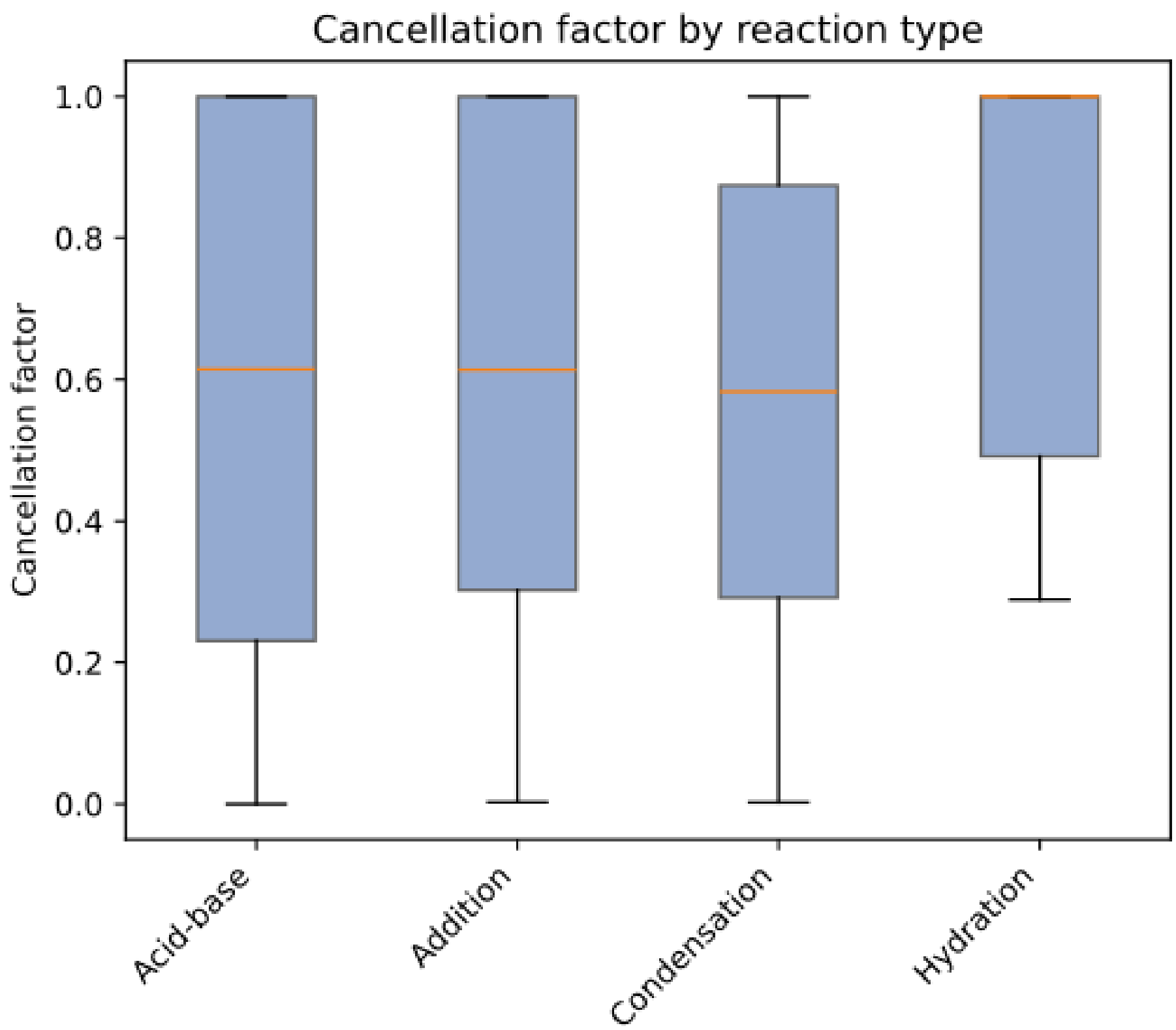

Figure SI XIII: Distribution of the cancellation factor grouped by reaction class. The cancellation factor is defined as $|\Delta G_{\rm err}|/\sum_i |e_i|$, where values approaching unity indicate little or no cancellation of molecular prediction errors.

8 Computational efficiency

8.1 Computational cost model

The principal motivation of POMs-GibbsNet is to eliminate the computational bottleneck associated with DFT vibrational frequency calculations while preserving the accuracy of Gibbs free-energy corrections. In a conventional quantum-chemical workflow, the total computational cost required to obtain molecular Gibbs free energies can be expressed as

$$T_{\mathrm{DFT}} = T_{\mathrm{opt}} + T_{\mathrm{freq}}, \tag{2}$$

where T_{opt} denotes the geometry optimization and T_{freq} corresponds to the vibrational frequency calculation used to obtain thermal free-energy corrections. Although geometry optimization is required irrespective of the thermochemical approach, frequency calculations become increasingly expensive for large polyoxometalate clusters because they require evaluation of the full Hessian matrix.

Using POMs-GibbsNet, the frequency calculation is replaced by a lightweight workflow consisting of descriptor generation, QTAIM analysis, and a single forward pass through the neural network. The corresponding computational cost becomes

$$T_{\mathrm{GibbsNet}} = T_{\mathrm{opt}} + T_{\mathrm{desc}} + T_{\mathrm{QTAIM}} + T_{\mathrm{ML}}, \tag{3}$$

where T_{desc}, T_{QTAIM}, and T_{ML} represent descriptor construction, QTAIM feature extraction, and neural-network inference, respectively. Since

$$T_{\mathrm{desc}} + T_{\mathrm{QTAIM}} + T_{\mathrm{ML}} \ll T_{\mathrm{freq}}, \tag{4}$$

the overall computational acceleration can be approximated as

$$S = \frac{T_{\mathrm{DFT}}}{T_{\mathrm{GibbsNet}}}. \tag{5}$$

The measured computational costs for the benchmark workflow are summarized in Table SI XVII. The DFT vibrational frequency calculation dominates the computational expense, whereas descriptor generation and GibbsNet inference contribute negligibly to the overall runtime.

Table SI XVII: Wall-clock computational cost of the GibbsNet workflow relative to a conventional DFT vibrational frequency calculation. The reported speedup is computed with respect to the DFT frequency calculation, which represents the computational bottleneck replaced by POMs-GibbsNet.

**Stage**	**Time (s)**	**Fraction of DFT cost**	**Relative speedup**
Geometry optimization	164.16	0.0690	14.5×
Descriptor generation	0.09	< 0.0001	27 596×
QTAIM analysis	1.88	0.0008	1 266×
GibbsNet inference	< 0.01	< 0.0001	$> 9 \times 10^6\times$
GibbsNet workflow (total)	166.12	0.0698	14.3×
DFT frequency calculation	2379.57	1.0000	1.0×

Overall, replacing the DFT frequency calculation with the POMs-GibbsNet workflow reduces the post-optimization computational cost by approximately one order of magnitude. While geometry optimization remains the dominant computational expense, the thermochemical correction becomes effectively negligible compared with the original vibrational analysis.

8.2 Scaling analysis

The computational advantage of POMs-GibbsNet is expected to increase further as molecular size increases. Conventional vibrational frequency calculations require evaluation and diagonalization of the molecular Hessian matrix, whose computational cost grows rapidly with the number of atoms. In contrast, descriptor generation, QTAIM feature extraction, and neural-network inference exhibit substantially more favorable scaling, depending primarily on local atomic environments and graph connectivity.

Consequently, although the benchmark reported in Table SI XVII already demonstrates an overall acceleration of approximately fourteen-fold, the relative advantage of POMs-GibbsNet is expected to become even greater for the larger polyoxometalate clusters that constitute the primary target systems of this work. Since the computational bottleneck shifts from vibrational analysis back to geometry optimization, the proposed workflow enables efficient thermochemical evaluation without modifying the underlying electronic-structure methodology.
